# A Programmable Nanovaccine Platform Based on M13 Bacteriophage for Personalized Cancer Vaccine and Therapy

**DOI:** 10.1002/adma.202510229

**Published:** 2025-08-27

**Authors:** Shengnan Huang, Yanpu He, Allison Madow, Huaiyao Peng, Mirielle Griffin, Jifa Qi, Mantao Huang, Heather Amoroso, Riley Abrashoff, Nimrod Heldman, Angela M. Belcher

**Affiliations:** ^1^ The David H. Koch Institute for Integrative Cancer Research Massachusetts Institute of Technology Cambridge MA 02139 USA; ^2^ Department of Biological Engineering Massachusetts Institute of Technology Cambridge MA 02139 USA; ^3^ Department of Brain and Cognitive Sciences Massachusetts Institute of Technology Cambridge MA 02139 USA; ^4^ Department of Nuclear Science and Engineering Massachusetts Institute of Technology Cambridge MA 02139 USA; ^5^ Biopolymers Core Lab The David H. Koch Institute for Integrative Cancer Research Massachusetts Institute of Technology Cambridge MA 02139 USA; ^6^ Department of Materials Science and Engineering Massachusetts Institute of Technology Cambridge MA 02139 USA

**Keywords:** biomaterials, cancer immunotherapy, M13 bacteriophage, nanovaccine, personalized cancer vaccine

## Abstract

Nanovaccines co‐assemble antigens and adjuvants to elicit robust immune responses but often require complex synthesis and post‐modification procedures. Here, a programmable nanovaccine platform based on the M13 bacteriophage is developed for the scalable production of vaccines and single‐step modular engineering of adjuvanticity, length, and antigen density. By reprogramming the sequence and size of the noncoding phage genome, the Toll‐like receptor 9 activation and the length of the phage are precisely controlled. With a novel molecular engineering approach, the antigen density is tuned from 13.6% to 70.3%. A systematic modulation reveals an optimal adjuvanticity at a constant antigen density for maximum anti‐tumor CD8^+^ T cell response, and vice versa, using the model antigen SIINFEKL. The M13 phage‐based nanovaccine induces durable memory immunity lasting over a year. In addition, a 24‐fold increase in neoantigen‐specific CD8^+^ T cell frequency is achieved when increasing both the adjuvanticity and antigen density. Furthermore, when combined with anti‐PD‐1 therapy, the M13 phage‐based personalized vaccine eradicates established MC‐38 tumors in 75% of treated animals and they develop 100% resistance against tumor invasion when challenged 5 months after treatment. These findings establish M13 phage as a powerful and versatile nanovaccine platform with transformative potential for personalized cancer immunotherapy.

## Graphical abstract

A reprogrammed (RP) phage‐based nanovaccine platform has been developed that allows single‐step assembly with precise control over adjuvanticity, antigen density, and nanoparticle size. By enhancing TLR9 activation and neoantigen display, this platform enables a potent personalized vaccine targeting tumor‐specific neoantigens. When combined with immune checkpoint inhibitors, the RP phage‐based vaccine induces tumor regression and provides complete protection against tumor rechallenge.

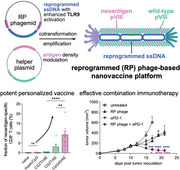

## 1 Introduction

Cancer vaccines represent a groundbreaking advancement in both cancer immunotherapy and disease prevention, offering transformative potential in the fight against cancer. Difficulties in the co‐delivery of antigens and adjuvants to secondary lymphoid organs, as well as the effective uptake of the vaccines by antigen‐presenting cells (APCs) remain major barriers to optimizing vaccine efficacy.^[^ ^1^ ^]^ To address these challenges, various nanovaccines, such as DNA origami, lipoprotein nanoparticle, and polymer nanoparticle, have been developed to co‐assemble the antigen and adjuvant; however, they often require multiple adsorption and conjugation steps.^[^ ^2^, ^3^, ^4^, ^5^ ^]^ Hence, approaches that could enable scalable single‐step synthesis would be attractive for clinical availability of vaccines. In this regard, the M13 bacteriophage stands out due to its scalable manufacturing, genetic manipulability, large antigen display area, favorable safety profile and high stability, making it highly suitable for clinical translations.^[^ ^6^, ^7^, ^8^, ^9^, ^10^, ^11^ ^]^ In particular, M13 phages have demonstrated superior biocompatibility toward mammalian cells and bacteriophages have been used to treat diseases in patients without notable adverse effects.^[^ ^11^, ^12^ ^]^

M13 phage is a type of filamentous bacteriophage composed of a single‐stranded DNA (ssDNA) encased in capsid proteins. It is ≈7 nm in diameter with a length determined by the ssDNA size, which can be controlled by a phagemid.^[^ ^13^ ^]^ M13 phage has been widely used in phage display to select proteins with high binding affinity toward the target substrates.^[^ ^13^, ^14^, ^15^ ^]^ For vaccine applications, its straightforward genetic system allows the display of significant quantities of antigen peptides on the major coat protein pVIII or minor coat protein pIII.^[^ ^8^, ^16^, ^17^, ^18^ ^]^ Additionally, the unmethylated cytosine‐phosphate‐guanine (CpG) motifs within the ssDNA genome activate Toll‐like receptor 9 (TLR9), contributing partially to its inherent adjuvanticity.^[^ ^19^, ^20^ ^]^ Current research on the M13 phage‐based vaccine focuses on modifying capsid proteins for antigen display and targeting with fixed length.^[^ ^21^, ^22^, ^23^, ^24^, ^25^ ^]^ The antibody response can be enhanced by doubling the antigen display via tandem antigen display on the phage capsid, highlighting the importance of antigen density for phage‐based vaccines.^[^ ^26^ ^]^ While the natural adjuvanticity of phage particles has been exploited, further efforts to enhance it may lead to more potent immune responses.^[^ ^27^, ^28^ ^]^ Furthermore, the size of nanovaccines plays a critical role in determining the lymph node drainage, APC uptake, and subsequent immune activation.^[^ ^29^ ^]^ These factors are critical for inducing strong immune responses, especially for weak antigens.^[^ ^30^, ^31^ ^]^ Inspired by this, we aim to advance the field of M13 phage‐based nanovaccines by modulating its antigen display, adjuvanticity, and length.

**Figure 1:**
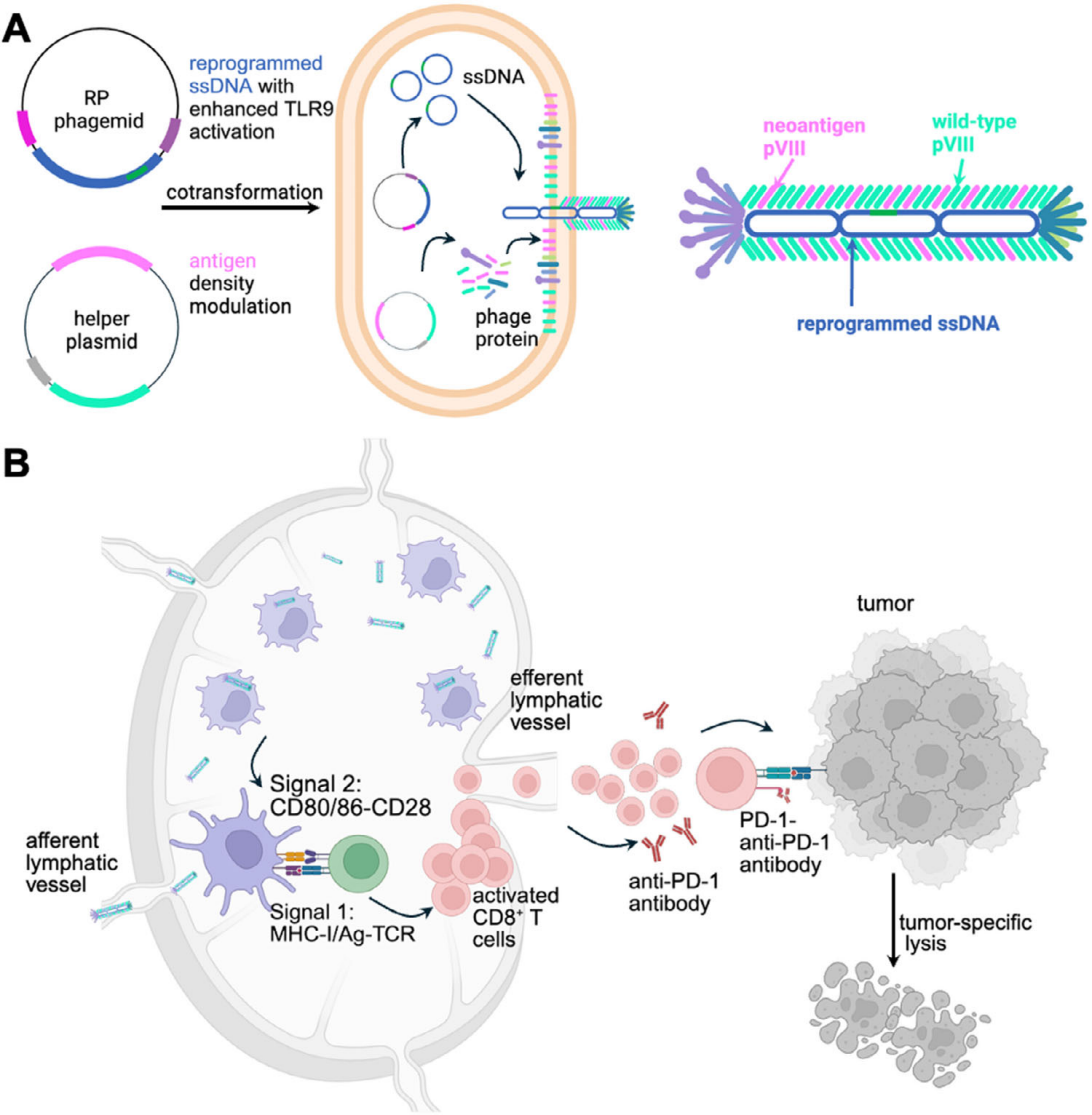
RP phage‐based vaccine for personalized cancer vaccine and therapy. A) RP phage amplification by co‐transforming the RP phagemid with reprogrammed ssDNA and the helper plasmid encoding the neoantigen pVIII. The sequence of the reprogrammed ssDNA is programmed to enhance the activation of the TLR9. The helper plasmid is designed to modulate the neoantigen pVIII display on the M13 phage capsid. B) RP phages are efficiently taken up by APCs in the lymph node, leading to strong antigen presentation (Signal 1) and APC maturation (Signal 2), which in turn present the antigen to the CD8^+^ T cells, resulting in the activation of antigen‐specific CD8^+^ T cells. The activated antigen‐specific CD8^+^ T cells recognize the cancer cells and eradicate the target cancer cells in the peripheral tissue. Combination with the immune checkpoint inhibitors, such as anti‐PD‐1 antibodies, further amplifies the potency of the RP phage‐based personalized vaccine for cancer immunotherapy.

In this study, we developed a programmable nanovaccine platform based on M13 phage, enabling single‐step modular engineering of adjuvanticity, length and antigen display (**Figure** **1**). We engineered a reprogrammed (RP) phage platform by programming its noncoding ssDNA sequence for precise regulation of TLR9 activation and phage length. In addition, we developed a novel molecular engineering approach to tune the antigen density. We modulated both the adjuvanticity and antigen density of the RP phage, revealing an optimal antigen density and adjuvanticity for maximum anti‐tumor CD8^+^ T cell response. Leveraging these insights, we developed an RP phage‐based personalized cancer vaccine targeting neoantigens (neoAgs). The RP phage vaccine elicited a remarkable 24‐fold increase in the frequency of neoAg‐specific CD8^+^ T cells, significantly inhibited tumor growth, and, when combined with immune checkpoint inhibitors (ICIs), eradicated established tumors in ≈75% of treated animals. These findings highlight the transformative potential of the RP phage platform as a promising strategy for personalized cancer vaccines and combinatorial immunotherapies, advancing the translational potential of virus‐based nanovaccines.

## 2 Results

### 2.1 Modulating the Adjuvanticity, Length and Antigen Density of the RP Phages

**Figure 2:**
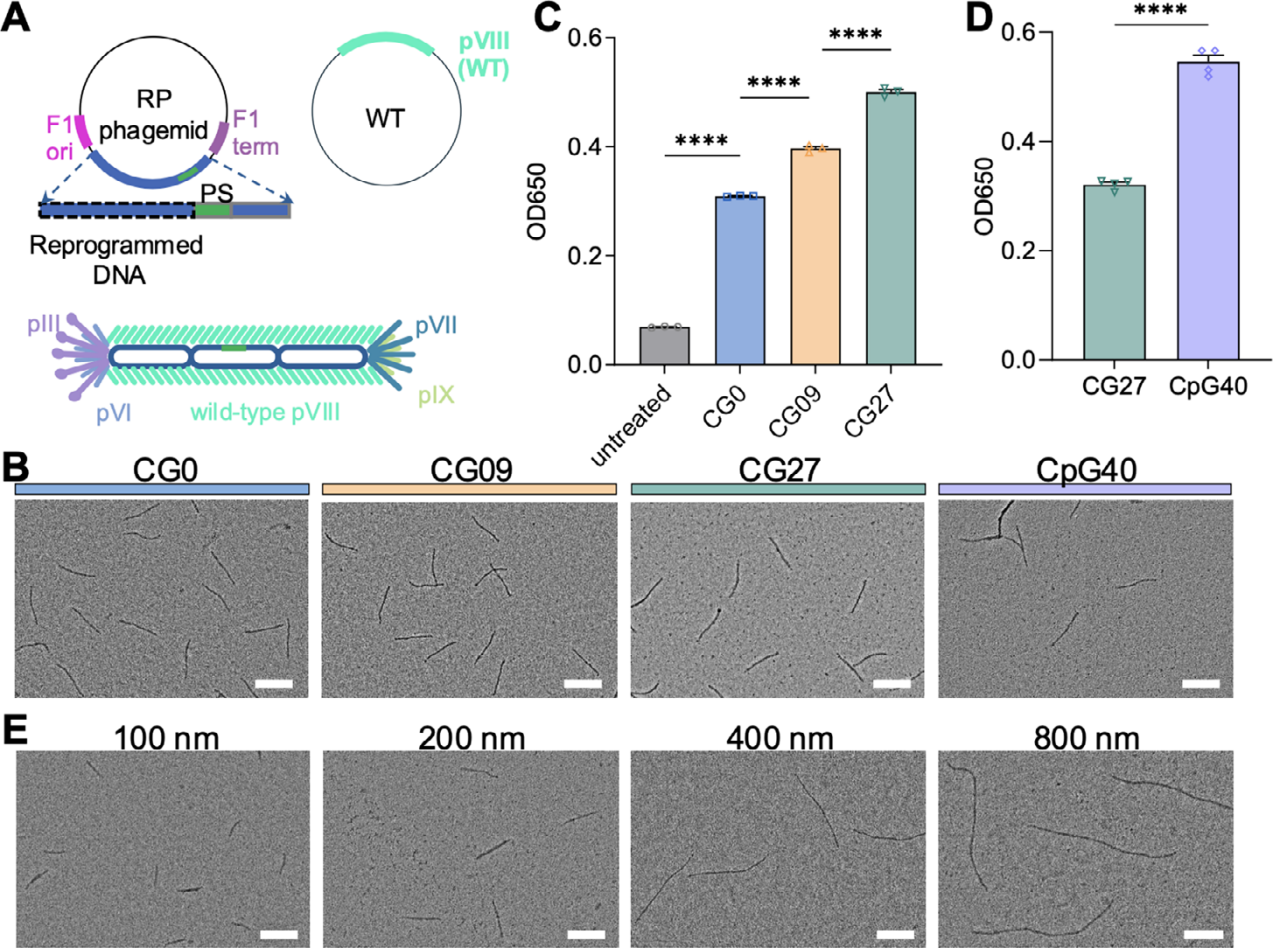
Modulating the adjuvanticity and length of the M13 phage by programming its ssDNA sequence. A) The ssDNA sequence in between the F1‐ori and F1‐term (black dashed outline) of the M13 phage was reprogrammed to increase its stimulation of the TLR9. B) Transmission electron microscopy (TEM) images of the RP phages showed the length of the RP phage was ≈200 nm. C) The activation of TLR9 increased with increasing CG fraction in the ssDNA when studied using the HEKBlue mTLR9 cell assay. D) CpG40 phagemid was developed when replacing 40% of the CG dimers in the CG27 phagemid with the canonical CpG hexamers. The as‐assembled RP phages exhibited higher TLR9 activation. E) RP phages of ≈100, 200, 400, and 800 nm were amplified. Scale bars represent 200 nm in (B) and (E). The data show mean ± s.e.m. from a representative experiment of 2‐3 independent experiments. ∗∗∗∗*p* < 0.0001, analyzed by one‐way ANOVA (C) with Bonferroni post hoc test, two‐tailed unpaired Student's *t*‐test (D).

The phagemid‐helper plasmid system was utilized to amplify RP phages, which enables precise and independent control over the sequence of the enclosed ssDNA (**Figure** **2**A).^[^ ^13^ ^]^ We aimed to enhance the adjuvanticity of the M13 phage by incorporating more CG dimers into the ssDNA while precisely controlling the inter‐CG ssDNA sequences to increase the TLR9 activation.^[^ ^32^, ^33^ ^]^ Specifically, we developed an algorithm (Methods section) to program the ssDNA sequence at single‐base level. We created a series of ssDNAs of the same size (1,415 nucleotides), with varying CG fractions of 0%, 9%, and 27%, designated as CG0, CG09, and CG27, respectively (refer to Table S1, Supporting Information for the detailed sequences). RP phages encapsulating the altered ssDNAs were amplified by combining the reprogrammed phagemids with a helper plasmid encoding the wild‐type (WT) pVIII. Since the length of the M13 phage is proportional to the ssDNA size, the RP phages developed were all ≈200 nm in length (Figure 2B), differing only in the ssDNA sequence. The HEKblue mouse TLR9 reporter cell line, which monitors secreted embryonic alkaline phosphatase via the TLR9‐dependent NF‐κB activation, was used to assess the ssDNA stimulation of the TLR9. The TLR9 activation was increased by ≈1.7‐fold (*p* < 0.0001) from CG0 to CG27 (Figure 2C, dose optimization study in Figure S1, Supporting Information). To further enhance TLR9 activation, we incorporated more potent canonical CpG hexamers (AACGTT and GACGTT) into the reprogrammed ssDNA.^[^ ^34^ ^]^ When mutating 40% of the CG dimers in CG27 into the CpG hexamers, CpG40 RP phages with a length of ≈220 nm were generated, which achieved ≈60% increase in TLR9 activation (Figure 2D, *p* < 0.0001) compared to the CG27 phages in an independent study, leading to an overall ≈2.7‐fold (CG0 vs. CpG40) increase in TLR9 activation. With the same programming rules, we developed a series of ssDNA sequences of varying length (721, 1447, 3241, and 6261 nucleotides), but similar CG fraction (≈25–27%, refer to Table S2, Supporting Information for the detailed ssDNA sequences and CG fractions). The lengths of the as‐assemble phages ranged from 100 nm to 800 nm (Figure 2E). Thus, we demonstrated that the adjuvanticity and length of M13 phage can be precisely modulated by programming its ssDNA sequence using our custom algorithm.

**Figure 3:**
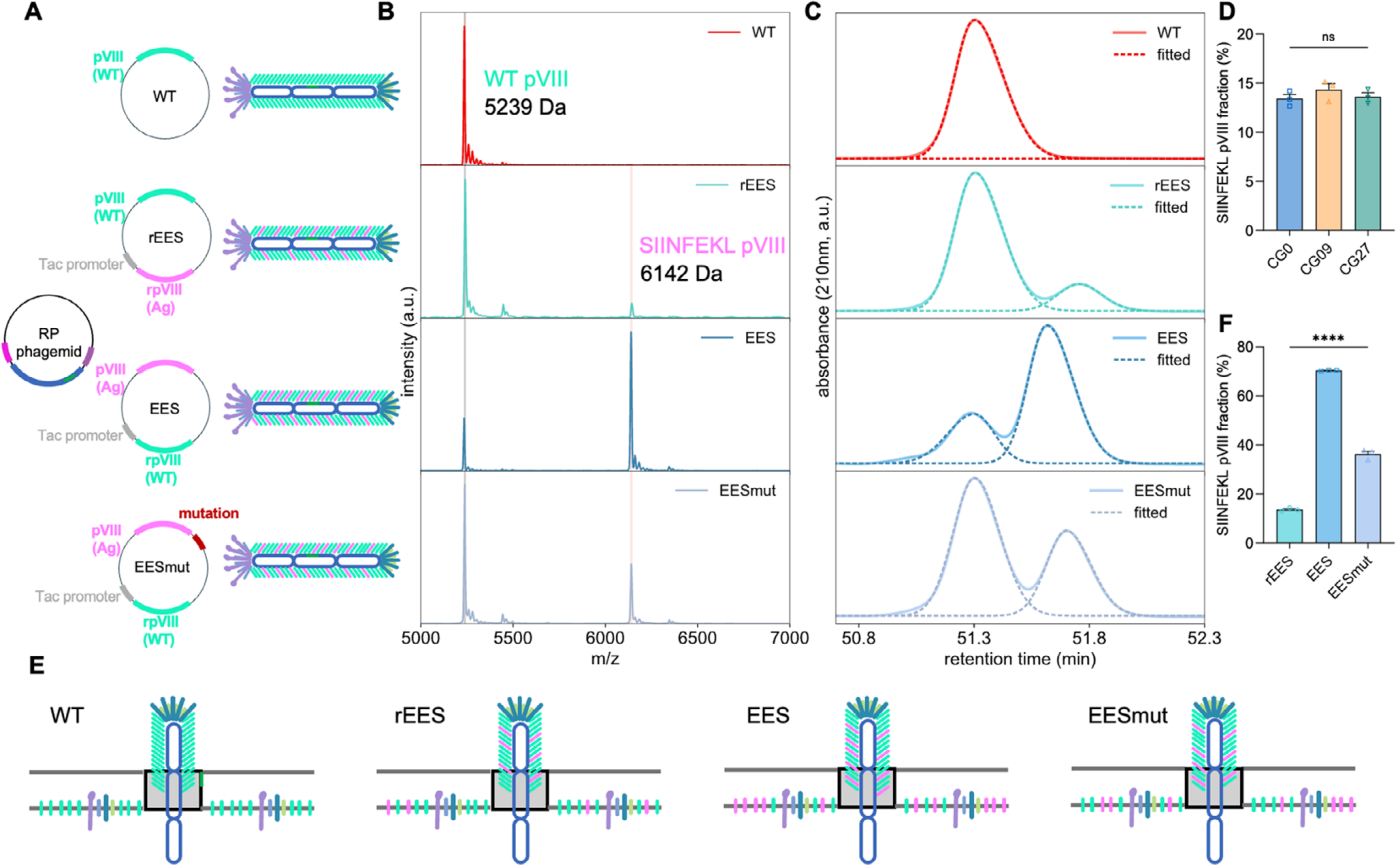
A novel approach to tuning the antigen density on the RP phage by controlling the proportion of antigen pVIII relative to wild‐type pVIII. A) SIINFEKL pVIII‐displaying RP phages were amplified by combining the RP phagemid and the SIINFEKL pVIII‐expressing helper plasmid: rEES, EES, and EESmut. B) Confirmation of the SIINFEKL pVIII display on the RP phage by MALDI‐TOF mass spectrometry. C) Quantification of the SIINFEKL pVIII display ratio on the RP phage by HPLC. D) SIINFEKL pVIII display ratio of RP phages amplified with various RP phagemids and the rEES helper plasmid was independent of the sequence of the phagemids (D). E) Illustration of controlling the SIINFEKL pVIII display on the RP phages. F) A summary of the SIINFEKL pVIII display ratios of RP phages amplified with various helper plasmids. The data show mean ± s.e.m. ns: not significant, ∗∗∗∗*p* < 0.0001, analyzed by one‐way ANOVA (D,F) with Bonferroni post hoc test.

For the initial vaccine studies, we tried to express SIINFEKL, a model antigen known to stimulate mouse CD8^+^ T cells through binding to the H‐2K^b^ of MHC‐I molecules, at the N‐terminus of pVIII, which is ideal for displaying antigen peptides in large quantities. In addition, as antigen peptides for the MHC‐I molecules are generally 8–10 amino acid residues long, pVIII N‐terminus display is suitable for displaying CD8^+^ T cell epitopes for cancer vaccine applications.^[^ ^35^ ^]^ We constructed a helper plasmid, rEES, containing a recombinant pVIII (rpVIII) under the control of a Tac promoter (**Figure** **3**A, second row) to express the SIINFEKL epitope with the peptide sequence of EESIINFEKL. The resulting M13 phage would be a mosaic phage consisting of WT and antigen pVIIIs, which was confirmed by matrix‐assisted laser desorption/ionization time‐of‐flight mass spectrometry (MALDI‐TOF MS, Figure 3B, first and second rows). The SIINFEKL pVIII peak at 6142 Da was detected along with the WT pVIII peak at 5239 Da. Next, we quantified the antigen pVIII display ratio (the proportion of antigen pVIIIs among all the pVIIIs) using high‐performance liquid chromatography (HPLC), which is more straightforward compared to the N‐terminal sequence analysis.^[^ ^36^, ^37^ ^]^ HPLC collection and MALDI‐TOF MS analysis (Figure S2, Supporting Information) confirmed that the HPLC peak at ≈51.3 min corresponded to the WT pVIII, while the shoulder peak following it represented the antigen pVIII (Figure 3C). The HPLC curve was fitted using a custom Double‐Gaussian fitting algorithm assuming that each HPLC peak could be modeled as a combination of two Gaussian distributions, and the antigen pVIII display ratio was quantified to be ≈13.6% (Figure 3C, second row), which was independent of the ssDNA sequence (Figure 3D) and length (Figure S2C, Supporting Information).

Then we aimed to increase the antigen display ratio, hypothesizing that higher antigen density could increase the antigen presentation to T cell receptors by APCs.^[^ ^30^, ^38^ ^]^ During M13 phage assembly, all the capsid proteins are synthesized in the *E. coli* cytoplasm and transported to the inner membrane for assembly, after which the phage is extruded from the pIV protein channel (Figure 3E).^[^ ^39^ ^]^ We hypothesized that increasing the proportion of antigen pVIIIs relative to the WT pVIIIs would increase their assembling probability, thereby increasing the antigen pVIII display ratio. To achieve this, we swapped the expression sites of WT and antigen pVIII in a new helper plasmid EES (Figure 3A, third row). MALDI‐TOF MS (Figure 3B, third row) and HPLC results (Figure 3C, third row) showed an increased display ratio of ≈70.3% (Figure 3F), however, the incorporation of antigen pVIIIs reduced the amplification yield. This could be explained by the interaction between the pVIIIs and the narrow pIV protein channel for phage extrusion during phage assembly.^[^ ^40^ ^]^ M13 phage has a fivefold symmetry and pVIIIs are assembled with multiple pentamer layers along the phage capsid. As more antigen pVIIIs are being assembled, the pVIII pentamer may experience transient increase in size and/or more disturbance to the interactions with the pIV channel,^[^ ^41^ ^]^ which will need additional adjustments in order to move forward to assemble the next pentamer layer. Hence, high antigen pVIII display could potentially compromise the phage amplification yield and we sought to identify any favorable mutations in the helper plasmid EES to increase the amplification yield without significantly compromising the SIINFEKL pVIII display ratio. By continuously monitoring the amplification yield and display ratio, a mutation preceding the start codon of the antigen pVIII was identified, resulting in the helper plasmid EESmut (Figure 3A, bottom row), which yielded an antigen pVIII display ratio of ≈36.3% (Figure 3B,C,F), while significantly enhancing the phage yield. To the best of our knowledge, this mutation, potentially a ribosome binding site mutation based on its location, has not been previously reported. The incorporation of the regulatory mutation may potentially decrease the synthesis rate of the SIINFEKL pVIII, thereby reducing its proportion on the inner membrane, leading to reduced SIINFEKL pVIII assembly into the phage capsid and lower SIINFEKL pVIII display. There is a tradeoff between the peptide length and its pVIII N‐terminus display ratio on the M13 phage.^[^ ^42^ ^]^ For a peptide of 10 amino acid residues (EESIINFEKL), a display ratio of ≈36.3% is considered high. Currently, three SIINFEKL pVIII antigen display ratios have been developed and further efforts could be done to expand the approach, for example, by using promoters of different strengths, to fine‐tune the antigen display ratio. In addition, the pVIII N‐terminus display approach developed in this study is aimed for displaying short CD8^+^ T cell epitopes at high surface density for cancer vaccine applications and may not be suitable for displaying long peptides, which may impact the immune activation due to the low surface density. In summary, we have developed a novel approach to control the antigen density, potentially enabling fine‐tuned modulation of the immune response based on the antigen density.

### 2.2 Potent CD8^+^ T Cell Response by the RP Phages

**Figure 4:**
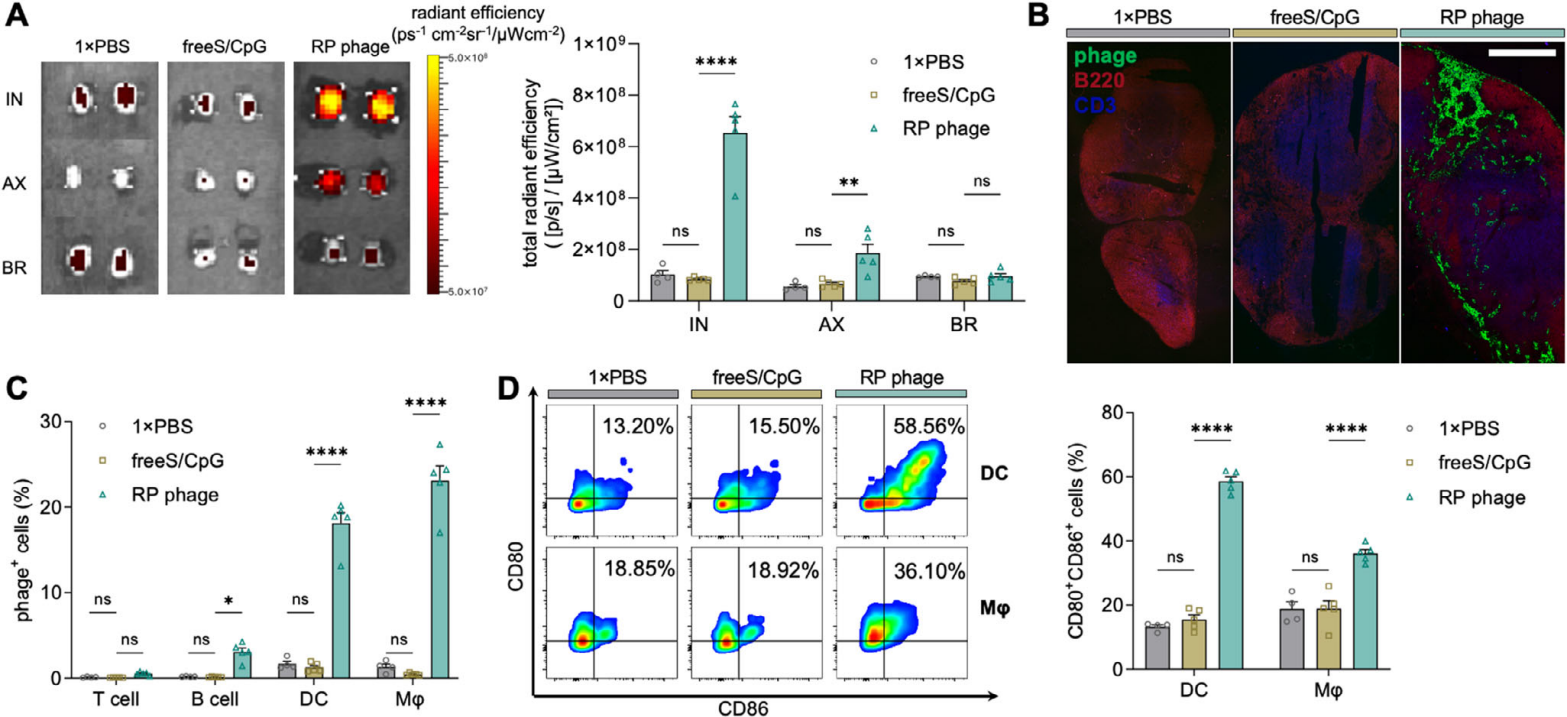
Accumulation of RP phages in the draining lymph nodes and professional APCs. A) RP phages (CG27/rEES) preferentially accumulated in the inguinal lymph node compared to free antigen as studied by the in vivo imaging system. IN: inguinal, AX: axillary, BR: brachial. B) Spatial distribution of RP phages in draining lymph nodes. Scale bar represents 500 µm. C) RP phages were mostly associated with macrophages (Mφs) and dendritic cells (DCs). D) Activation of Mφs and DCs by RP phages as indicated by the upregulation of CD80 and CD86. The data labeled is the mean of each group. The data show mean ± s.e.m. from 2‐3 independent experiments (*n* = 4‐5). ns: not significant, ∗*p* < 0.05, ∗∗*p* < 0.01, and ∗∗∗∗*p* < 0.0001, analyzed by two‐way ANOVA (A,C,D) with Bonferroni post hoc test.

As antigen presentation and T cell priming orchestrate in lymph nodes (LNs),^[^ ^43^ ^]^ we next characterized the RP phage (≈200 nm long) drainage into the draining LNs (dLNs) by conjugating it with fluorescein (Figure S3, Supporting Information). 24 h after the subcutaneous injection at the tail base, RP phages showed significantly greater accumulation in inguinal (IN) LNs than the free antigen and adjuvant combinations (free SIINFEKL and CpG, freeS/CpG, **Figure** **4**A, *p* < 0.0001), while freeS/CpG showed minimal LN drainage. RP phages also tended to accumulate in the axillary (AX) LNs (≈20% of IN LNs) with minimal accumulation in the brachial (BR) LNs. Similarly, histological sections of the dLNs revealed minimal detectable free antigen, while RP phages accumulated in the subcapsular sinus and interfollicular areas (Figure 4B). The enhanced LN trafficking of RP phages could be attributed to their nanoscale dimensions, which exploit the physiological characteristics of lymphatic drainage.^[^ ^44^ ^]^ Within the immune cell populations of the dLNs, RP phages were mostly associated with macrophages (Mφs, CD3^−^/B220^−^/CD11b^+^/CD11c^−^/F4/80^+^, ≈23.1%), dendritic cells (DCs, CD3^−^/B220^−^/CD11b^−^/CD11c^+^, ≈18.1%) and B cells (CD3^−^/B220^+^, ≈3.1%) as in Figure 4C, all of which are professional APCs (FACS gating approach in Figure S4, Supporting Information), consistent with refs. [43, 45]. The preferential accumulation of the RP phages within Mφs and DCs could be ascribed to their size (7 nm in diameter, 200 nm in length), which could facilitate the endocytosis possibly mediated by clathrin and caveolin receptors.^[^ ^29^ ^]^ Importantly, RP phages led to the activation of Mφs and DCs, as indicated by the elevated expression of co‐stimulatory molecules CD80 and CD86 (Figure 4D). Compared to the freeS/CpG group, the RP phage group exhibited a ≈four fold increase in CD80^+^CD86^+^ DCs (*p* < 0.0001) and a ≈two fold increase in CD80^+^CD86^+^ Mφs (*p* < 0.0001), while freeS/CpG had minimal effect on Mφ and DC maturation compared to 1× PBS. Overall, the results suggested that RP phages efficiently drained to the dLNs, preferentially accumulated in professional APCs, and substantially enhanced their activation, thereby providing the foundation for effective antigen presentation and subsequent T‐cell activation in the dLNs.

**Figure 5:**
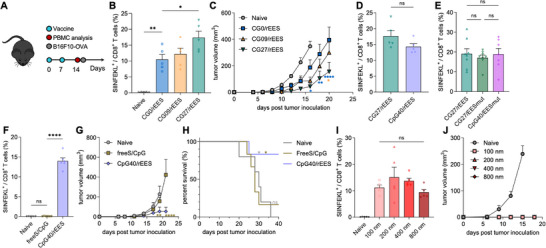
Optimum antigen density and adjuvanticity for maximum anti‐tumor CD8^+^ T cell response studied using the RP phage platform. A) Vaccination dosing schedule of the RP phages in C57BL/6 mice. Two doses of RP phages were administered via tail base injection on day 0 and day 7. Peripheral blood was analyzed on day 14 for SIINFEKL‐specific CD8^+^ T cells and B16F10‐OVA cells were inoculated on day 15. B) The frequency of SIINFEKL‐specific CD8^+^ T cells increased with increasing adjuvanticity of the RP phages amplified by the same helper plasmid (rEES) and various phagemids (CG0/09/27). C) On day 15, mice were challenged with 0.5M B16F10‐OVA cells subcutaneously on the left flank, and tumor growth was monitored over time. D) When increasing the adjuvanticity of the RP phages (CG27 vs CpG40), the fraction of SIINFEKL‐specific CD8^+^ T cells did not increase. E) Increasing antigen density (rEES vs EESmut) and adjuvanticity (CG27 vs CpG40) did not enhance the SIINFEKL‐specific CD8^+^ T cell response. F‐H) RP phage demonstrated better vaccination efficacy compared to free antigen and adjuvant. (F) Free SIINFEKL peptide and CpG formulation (freeS/CpG) elicited minimal T‐cell response. RP phages significantly slowed down the tumor progression (G) and prevented tumors from developing with prolonged survival (H) compared to freeS/CpG when challenged with 0.2M B16F10‐OVA cells. I,J) The frequency of SIINFEKL‐specific CD8^+^ T cells depended little on the length of the RP phages (I) and vaccination with RP phages prevented tumors from developing when challenged with 0.3M B16F10‐OVA cells on day 15 (J). The data show mean ± s.e.m. ns: not significant, ∗*p* < 0.05, ∗∗*p* < 0.01, and ∗∗∗∗*p* < 0.0001, analyzed by one‐way ANOVA (B,E,F,I) or two‐way ANOVA (C,G) with Bonferroni post hoc test, two‐tailed unpaired Student's *t*‐test (D), and log rank test (H).

We then studied the impact of antigen density and adjuvanticity on the nanovaccine efficacy by using the versatile RP phage platform. Female C57BL/6 mice were immunized with RP phages of varying adjuvanticity (CG0, CG09, CG27, Figure 2C) but with the same ssDNA size (Figure 2B) and antigen density (rEES, Figure 3D) (**Figure** **5**A). The vaccination dose was determined based on the adjuvant and antigen dose in relevant nanovaccine works.^[^ ^2^, ^46^, ^47^ ^]^ The results showed a marked increase in the frequency of SIINFEKL‐specific CD8^+^ T cells with increasing adjuvanticity, from ≈10.5% to ≈17.4% (CG0 vs CG27, *p* < 0.05, Figure 5B). Upon challenge with B16F10‐OVA cells, mice vaccinated with RP phages of CG27/rEES showed slower tumor growth compared to other groups (Figure 5C). However, further increasing the adjuvanticity without changing the antigen density (CG27/rEES vs CpG40/rEES) did not boost the vaccine efficacy (Figure 5D). Overall, these results demonstrated that at a constant antigen density (13.6% antigen pVIII display ratio), a maximum CD8^+^ T cell response was achieved with the CG27 phagemid. On the other hand, at constant adjuvanticity (CG27), the SIINFEKL‐specific CD8^+^ T cell frequency was decreased when decreasing the antigen pVIII display ratio below 13.6% (Figure S5, Supporting Information), whereas minimal enhancement in the anti‐tumor CD8^+^ T cell response was observed when increasing the antigen pVIII display ratio to 36.3% (CG27/rEES vs CG27/EESmut, Figure 5E). These results suggested that at constant adjuvanticity (CG27), maximum anti‐tumor CD8^+^ T cell response was achieved at an antigen pVIII display ratio of 13.6%. A similar CD8^+^ T cell activation plateau has also been observed at elevated antigen concentrations when stimulating antigen‐specific CD8^+^ cells in vitro.^[^ ^48^ ^]^ This could be ascribed to a variety of reasons, such as the limitations in antigen processing and presentation efficiency, MHC‐I presentation saturation on the APC surface, T‐cell receptor activation saturation, desensitization of T‐cell receptor signaling, or immune tolerance.^[^ ^49^, ^50^ ^]^ Furthermore, increasing both the adjuvanticity and antigen density (CG27/rEES vs. CG27/EESmut vs. CpG40/EESmut, Figure 5E) did not boost the antigen‐specific CD8^+^ T cell response. Collectively, these results indicated that at constant antigen density, there is an optimal adjuvanticity for maximum CD8^+^ T cell responses and vice versa. Additionally, free S/CpG induced minimal SIINFEKL‐specific CD8^+^ T cell response and provided no protection against tumor invasion when challenged with B16F10‐OVA cells (Figure 5F,G). In contrast, 5 out of 6 mice in the RP phage groups remained tumor‐free 40 days post‐inoculation (Figure 5H).

Then RP phages of varying length (Figure 2E) but with the same CG fraction in the ssDNA genome, the same antigen display, and similar zeta potential and stability (Figure S6, Supporting Information) were used to study whether the immune response could be modulated by controlling the length of the RP phages. RP phages elicited strong antigen‐specific CD8^+^ T cell responses (Figure 5I), effectively protecting 100% of the mice (5 out of 5) from developing tumors in the 200, 400, 800 nm groups and 80% of mice (4 out of 5) in the 100 nm group (Figure 5J). Surprisingly, the vaccine efficacy depended little on the length of the RP phages. This observation could be attributed to several factors. One possible reason could be the different draining kinetics of the phages of varying length. Shorter phages demonstrated higher lymph node draining rates than longer phages (Figure S7, Supporting Information), indicating that longer phage fibers could potentially act as a vaccine reservoir at the injection site for sustained release of vaccines over time, which could contribute to their vaccine efficacy.^[^ ^51^ ^]^ Another possible explanation could be that all the phages (100–800 nm in Figure 5J) delivered an antigen dose that reached or surpassed the optimal antigen dose for the maximum CD8^+^ T cell response. To better understand the length effect, we decreased the antigen pVIII display ratio to ≈6%, and the 100 nm phages demonstrated a higher antigen‐specific CD8^+^ T cell response than the 800 nm phages (Figure S8, Supporting Information).

**Figure 6:**
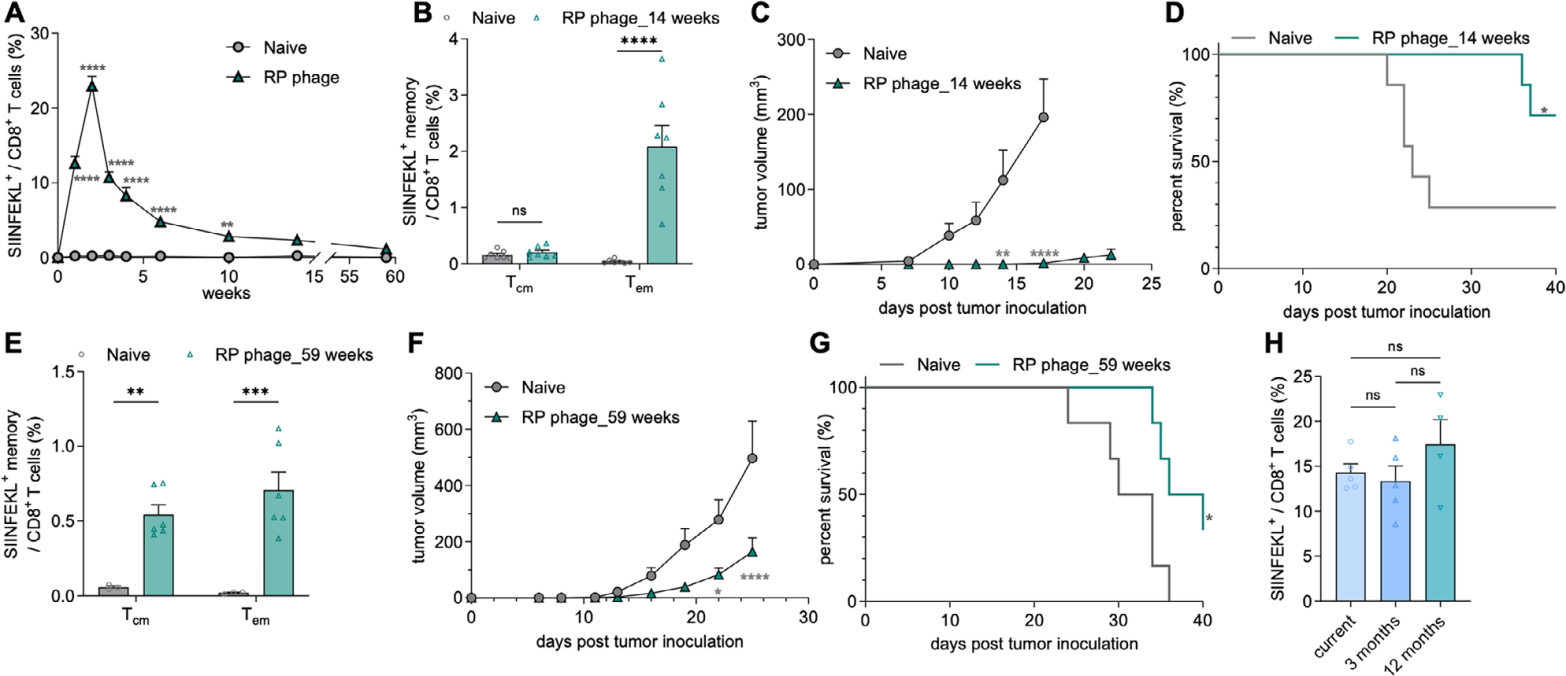
Robust and long‐lasting memory CD8^+^ T cell response elicited by RP phages. A) Fraction of SIINFEKL‐specific CD8^+^ T cells over time post RP phage administration on day 0 and day 7. B‐D) SIINFEKL‐specific CD8^+^ T cell memory study at week 14. (B) Fraction of SIINFEKL‐specific central memory CD8^+^ T cells (T_cm_) and effector memory CD8^+^ T cells (T_em_) at week 14. Vaccination with RP phages slowed down tumor progression (C) and prevented tumors from developing (D) for mice at week 14. E‐G) SIINFEKL‐specific CD8^+^ T cell memory study at week 59. (E) Fraction of T_cm_ and T_em_ at week 59 in an independent study. Vaccination with RP phages slowed down tumor progression (F) and extended the survival (G) of mice at week 59. H) RP phages showed comparable vaccination efficacy after 1 year of storage at 4 °C. The data show mean ± s.e.m. ns: not significant, ∗*p* < 0.05, ∗∗*p* < 0.01, ∗∗∗*p* < 0.001 and ∗∗∗∗*p* < 0.0001, analyzed by one‐way ANOVA (H) or two‐way ANOVA(A,B,C,E,F) with Bonferroni post hoc test and log rank test (D,G).

We then examined the durability of the antigen‐specific CD8^+^ T cells elicited by RP phages. The frequency of the SIINFEKL‐specific CD8^+^ T cells first expanded before contracting, peaking one week after the second dose (**Figure** **6**A). By week 14, the frequency reached ≈2.3%, decreasing to ≈1.2% by week 59 in an independent study. The temporal response of the antigen‐specific CD8^+^ T cells elicited by RP phages resembles a typical T cell response pattern post antigen stimulation with expansion, contraction, and memory phases.^[^ ^52^ ^]^ At week 14, phenotype analysis of the SIINFEKL‐specific CD8^+^ T cells showed that ≈90% (2.1% out of 2.3%) were effector memory T cells (T_em_, CD8^+^/SIINFEKL^+^/CD44^+^/CD62L^−^) with negligible central memory T cells (T_cm_, CD8^+^/SIINFEKL^+^/CD44^+^/CD62L^+^) (Figure 6B, FACS gating strategy in Figure S9, Supporting Information). Upon challenge with B16F10‐OVA cells, RP phages significantly slowed down the tumor progression compared to age‐matched naïve mice, with 5 out of 7 mice remaining tumor‐free on day 40 (Figure 6C,D). By week 59, phenotype analysis of the SIINFEKL‐specific CD8^+^ T cells revealed a significant shift with ≈42% T_cm_ (0.5% out of 1.2%) and ≈58% T_em_ (0.7% out of 1.2%) (Figure 6E), indicating durable CD8^+^ T cell memory and phenotypic evolution over time.^[^ ^53^ ^]^ Upon inoculation with B16F10‐OVA cells, the long‐lasting SIINFEKL‐specific memory CD8^+^ T cells effectively delayed tumor progression (Figure 6F,G). Additionally, RP phages stored at 4 °C for one year elicited comparable antigen‐specific CD8^+^ T cell response as the freshly prepared RP phages (Figure 6H), underscoring the exceptional stability of RP phages, which is critical for broad vaccine distribution. Moreover, we demonstrated that the phages exhibited superior biocompatibility toward mammalian cells (Figure S10, Supporting Information), together with the comprehensive in vivo mouse toxicity studies by Yue et al. and Tsedev et al.^[^ ^6^, ^13^ ^]^ These are promising data that ease the safety concern of M13 phages for biomedical applications. In summary, we have developed a highly uniform and stable nanovaccine platform that displays robust CD8^+^ T cell activation, enabling single‐step assembly at a potentially low production cost.

### 2.3 RP Phage‐Based Personalized Cancer Vaccine for Combined Immunotherapy

**Figure 7:**
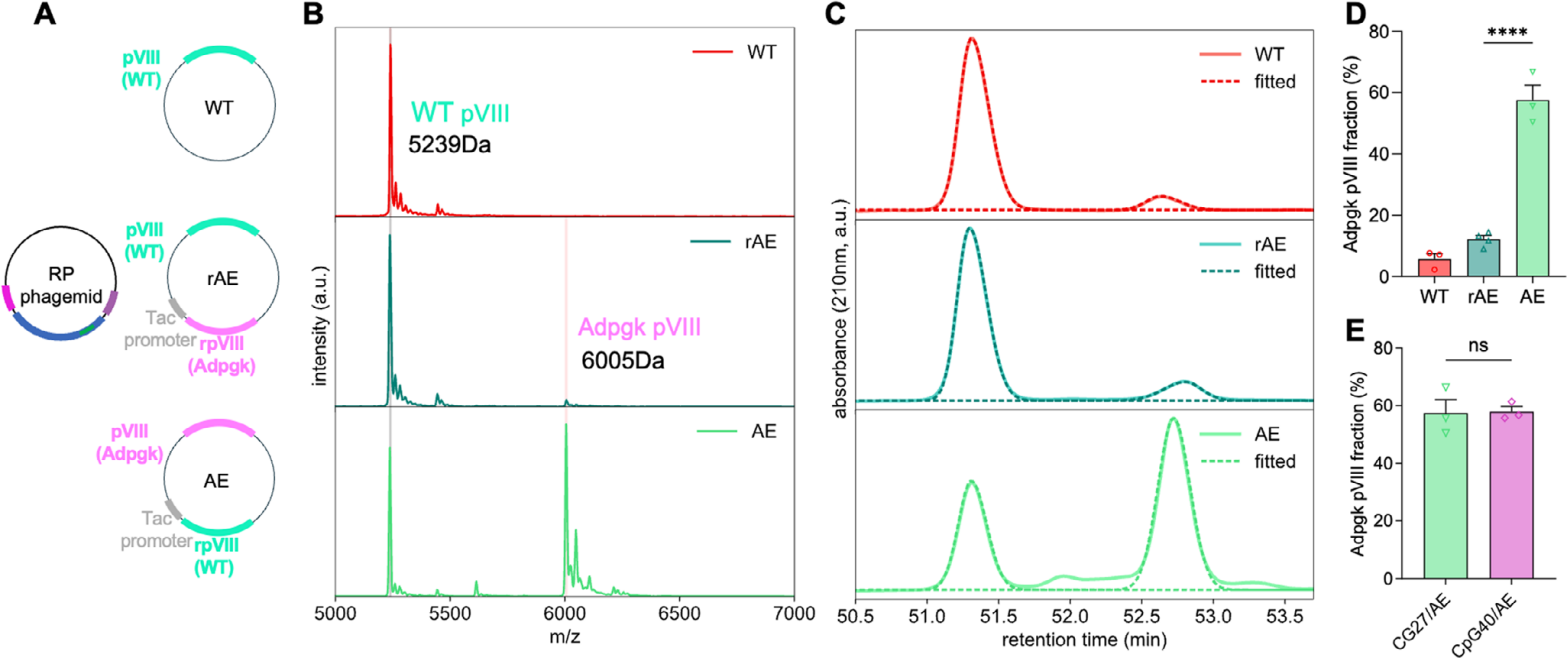
Tuning the neoantigen pVIII display ratio on RP phages. A) Adpgk pVIII‐displaying RP phages amplified with the RP phagemid and the helper plasmids: rAE and AE. B‐E) Quantification of the Adpgk pVIII display ratio. Confirmation and quantification of the Adpgk pVIII with MALDI‐TOF (B) and HPLC (C), respectively. (D) Adpgk pVIII display ratio for the rAE and AE helper plasmids. (E) The Adpgk pVIII display density was independent of the phagemids. The data show mean ± s.e.m. ns: not significant, ∗∗∗∗*p* < 0.0001, analyzed by one‐way ANOVA with Bonferroni post hoc test (D) and two‐tailed unpaired Student's *t*‐test (E).

To demonstrate the versatility and translational potential of our RP phage platform for personalized cancer vaccines targeting neoAgs, we engineered RP phages displaying the Adpgk neoAg in the MC‐38 colon carcinoma model. The Adpgk neoAg harbors a single epitope mutation (ASMTNRELM → ASMTNMELM) and is known to be presented by the MHC‐I H‐2D^b^ molecules.^[^ ^2^, ^46^ ^]^ The presence of the Adpgk mutation in the MC‐38 cell was confirmed with cDNA sequencing (Figure S11, Supporting Information). Utilizing the antigen pVIII density modulation approach developed in Figure 3, we tuned the Adpgk pVIII density on the RP phages with the helper plasmids, rAE and AE (**Figure** **7**A). Specifically, MALDI‐TOF MS verified the presence of the Adpgk pVIII peak at 6005 Da, with a notable increase with the AE helper plasmid (Figure 7B). HPLC (Figure 7C; Figure S12, Supporting Information) and the Double‐Gaussian fitting method estimated the Adpgk pVIII display ratios at ≈12.2% and ≈57.4% for rAE and AE plasmids, respectively (Figure 7D), which was also independent of the phagemids (Figure 7E). The yield of the RP phage with the AE helper plasmid was sufficient for the in vivo vaccination study, and the regulatory mutation in Figure 3 could also be applicable to further tune the neoantigen density. The results show that the systematic approach to tuning the antigen pVIII display ratio developed in this study can be generalized to other antigen peptides of interest.

**Figure 8:**
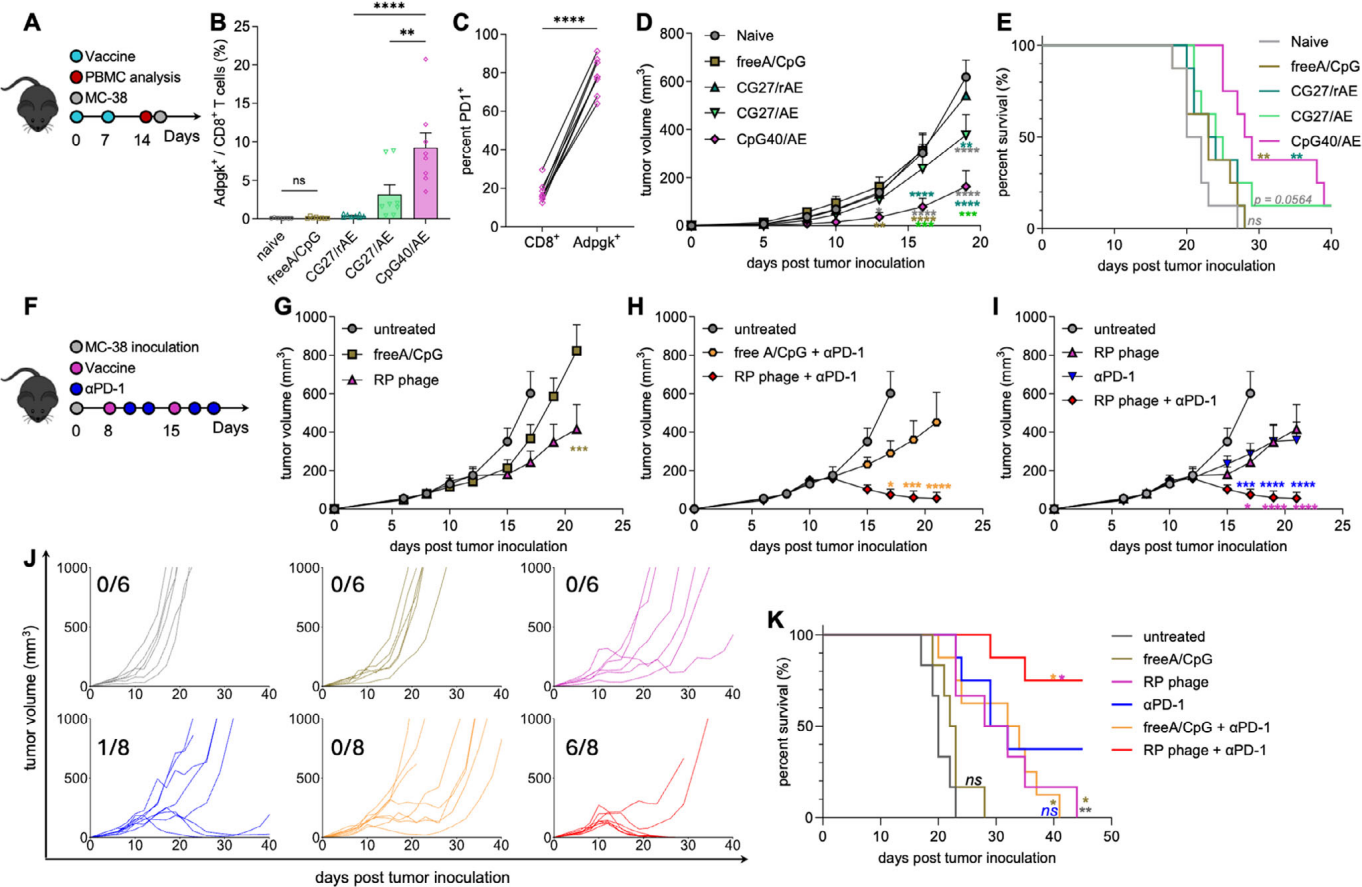
RP phage‐based personalized cancer vaccine eradicated established colon cancer when combined with immune checkpoint inhibitors. A–E) Enhancing RP phage vaccination efficacy by increasing the adjuvanticity and neoantigen density of RP phages. (A) Dosing schedule of the vaccination study. (B) Increasing both the Adpgk pVIII display and adjuvanticity of the RP phage led to stronger Adpgk‐specific CD8^+^ T cell response. (C) Upregulation of the PD‐1 expression in the Adpgk‐specific CD8^+^ T cells. Vaccination with the RP phage of CpG40/AE slowed down the MC‐38 tumor growth (D) and prolonged the survival (E) of the mice. F‐K) Strong synergy between the RP phage‐based personalized vaccine and anti‐PD‐1 therapy led to complete tumor regression. (F) C57BL/6 mice were subcutaneously inoculated with 0.25M MC‐38 cells on day 0. For vaccine only treatment, mice were vaccinated with the RP phage or soluble vaccine via tail base injection on days 8 and 15. For anti‐PD‐1 antibody treatment only, mice were administered with anti‐PD‐1 antibodies intraperitoneally on days 10, 12, 17, and 19. For combined therapy, on day 2 and 4 after each vaccination, mice were administered intraperitoneally with anti‐PD‐1 antibodies. Average tumor volume of the vaccine only treatment study (G), combined therapy study (H), and synergy study between RP phages and anti‐PD‐1 antibodies (I). Individual MC‐38 tumor growth curves (J) and survival curves (K) of the different study groups in (G, H, I). All the tumor treatment data were from the same cohort of studies. The data show mean ± s.e.m. from 2‐3 independent experiments (n = 6‐8). ns: not significant, ∗*p* < 0.05, ∗∗*p* < 0.01, ∗∗∗*p* < 0.001 and ∗∗∗∗*p* < 0.0001, analyzed by two‐tailed unpaired Student's *t*‐test (C), one‐way ANOVA (B) or two‐way ANOVA (D,G,H,I) with Bonferroni post hoc test and log rank test (E, K).

In a preliminary vaccination trial following the schedule in **Figure** **8**A, RP phages of varying adjuvanticity but the same antigen density (CG0/rAE, CG09/rAE, CG27/rAE, and CpG40/rAE) elicited low Adpgk‐specific CD8^+^ T cell frequencies (Figure S13, Supporting Information), suggesting that antigen density might be the limiting factor for robust anti‐tumor CD8^+^ T cell response. To address this, we used the AE plasmid to increase the Adpgk pVIII display ratio. In vivo vaccination results demonstrated that the RP phage CpG40/AE exhibited a significant increase in Adpgk‐specific CD8^+^ T cell fraction of ≈9.3%, representing a ≈24‐fold enhancement compared to the CG27/rAE (≈0.38%, *p* < 0.0001, Figure 8B). Comparing the in vivo vaccination efficacy of the SIINFEKL‐displaying RP phages (Figure 2D,E) and that of the Adpgk‐displaying RP phages demonstrated that the SIINFEKL‐displaying phages achieved maximal CD8^+^ T cell activation at a lower antigen pVIII display ratio (13.6%) and lower adjuvanticity (CG27), with minimal enhancement when further increasing the adjuvanticity (CpG40). However, when displaying the Adpgk pVIII at a ratio of 57.4%, the CD8^+^ T cell response can still be improved with higher adjuvanticity (CG27 vs CpG40, Figure 8D). Considering the antigen presentation process (Figure 1), this could be explained by a variety of mechanisms. One possible mechanism could be the different peptide‐MHC T cell receptor (pMHC‐TCR) binding affinity for SIINFEKL and the neoantigen Adpgk. SIINFEKL, as a foreign peptide, may exhibit higher pMHC‐TCR binding affinity compared to that of the tumor antigen Adpgk.^[^ ^54^, ^55^, ^56^ ^]^ The lower pMHC‐TCR binding affinity of Adpgk may be compensated by higher antigen dose and stronger adjuvanticity in order to elicit potent CD8^+^ T cell activation. In addition, the PD‐1 receptor expression was upregulated on the Adpgk‐specific CD8^+^ T cells compared to the total CD8^+^ T cells, a phenomenon also observed by other cancer nanovaccine studies (Figure 8C).^[^ ^46^, ^47^ ^]^ The expression of PD‐1 on CD8^+^ T cells is correlated with the antigen‐T cell receptor signaling, and thus with the functional avidity of the antigen‐specific CD8^+^ T cells.^[^ ^57^, ^58^, ^59^ ^]^ The upregulation of the PD‐1 on CD8^+^ T cells is also an approach adopted by the immune system to avoid autoimmunity.^[^ ^60^ ^]^ However, this pathway is hijacked by the tumor cells to evade peripheral surveillance through the PD‐1/PD‐L1 pathway. To block this pathway, immune checkpoint blockade therapies have been developed and demonstrated better treatment outcomes in various cancer types. Furthermore, when challenged with MC‐38 cells on day 15, CpG40/AE significantly delayed the tumor progression (Figure 8D), and improved survival compared to the other study groups (Figure 8E). Specifically, CpG40/AE prolonged survival by ≈40% relative to the naïve group (median survival 29.5 days vs 21 days, *p* < 0.001). In contrast, vaccination with free neoantigen and adjuvant (free A/CpG) failed to elicit Adpgk‐specific CD8^+^ T cells and inhibit the tumor progression compared to the naïve group. Thus, we developed an RP phage‐based personalized cancer vaccine that demonstrated strong prophylactic efficacy by enhancing the neoAg pVIII density and adjuvanticity of the phage. The greatly enhanced neoantigen‐specific CD8^+^ T cell response highlights the significance of the systematic approach for modulating antigen display. The approach can be applied to develop a robust personalized cancer vaccine and therapy based on the M13 phage, targeting patient‐derived neoantigens.

Lastly, we evaluated the therapeutic efficacy of the RP phage‐based personalized cancer vaccine. Following the treatment schedule in Figure 8F, the RP phage (CpG40/AE) significantly inhibited the tumor growth, compared to other study groups (Figure 8G) with a median survival of 30 days, representing an increase of ≈50% and ≈33% than the untreated group (20 days) and soluble vaccine group (22.5 days), respectively. However, RP phage treatment did not result in complete tumor regression, likely due to the immunosuppression within the tumor microenvironment.^[^ ^2^, ^61^ ^]^ In order to block the immune‐suppressive PD‐1/PD‐L1 pathway, we combined the RP phage vaccine with anti‐PD‐1 (*α*PD‐1) antibodies for immune checkpoint blockade (ICB) therapy.^[^ ^62^ ^]^ We found that the combination therapy efficacy depended critically on the treatment timing (Figure S14, Supporting Information), highlighting the importance of early tumor detection for achieving better treatment outcomes.^[^ ^63^ ^]^ When initiating the treatment on day 8 with an average tumor size of ≈80 mm^3^, although the RP phage and *α*PD‐1 alone demonstrated similar treatment efficacy, the combination significantly enhanced the treatment outcomes (Figure 8H) and demonstrated strong synergy (Figure 8I), achieving complete tumor regression in ≈75% of mice (6 out of 8, Figure 8J,K). The combination therapy may alter the immune cell populations within the tumor microenvironment with more effector CD8^+^ T cells, neoantigen‐specific CD8^+^ T cells, type 1 helper CD4^+^ T cells and proinflammatory macrophages, shifting the tumor microenvironment from immunosuppressive to immunoactive state.^[^ ^2^, ^47^, ^64^, ^65^, ^66^ ^]^ In addition, the mice in different treatment groups experienced little body weight loss (Figure S15, Supporting Information) during the course of the treatment, indicating that the combination therapy posed little toxicity to the mice. However, one should be aware that due to the PD‐1/PD‐L1 pathway blockage during the course of ICB treatment, normal tissues may get damaged manifesting as immune‐related adverse effects, which could be further studied with a variety of indicators, for example, the biomarkers associated with cellular damage.^[^ ^67^ ^]^ About 5 months post combination therapy, the mice with complete tumor regression in the RP phage + *α*PD‐1 group were challenged with MC‐38 tumor cells, none of them developed tumors 50 days post tumor inoculation (Figure S16, Supporting Information). On the contrary, the age‐matched naïve mice all succumbed to tumor burden, suggesting the strong memory immunity developed by the combination therapy. Overall, the RP phage‐based personalized cancer vaccine shows great potential for therapeutic treatment of cancer, particularly when combined with other treatment modalities.

The optimal antigen density and adjuvanticity necessary for effective personalized cancer therapy vary among different antigens and thus should be independently evaluated and optimized. One important parameter to facilitate the evaluation could be the immunogenicity of the antigen. The immunogenicity could be assessed by parameters such as the binding affinity between the antigen and the MHC, the stability of the pMHC complex, and the binding affinity between the pMHC and the T cell receptor, which can be predicted by well‐developed algorithms, such as NetMHCpan and DeepImmuno.^[^ ^68^, ^69^ ^]^

## 3 Conclusion

In summary, the study successfully demonstrates the potential of the RP phage as a versatile and effective nanovaccine platform by optimizing both its adjuvanticity and antigen density. The novel algorithm developed for reprogramming the genome and the innovative approach to tune the antigen density have provided valuable insights into vaccine design. Using SIINFEKL as a model antigen, we showed that enhancing the adjuvanticity of the M13 phage could significantly boost the immune response; however, there was an optimum dose for both antigen and adjuvant for maximum vaccine efficacy. Leveraging these insights in developing a personalized cancer vaccine targeting the MC‐38 neoantigen Adpgk highlights the practical benefits of this approach, leading to drastically enhanced T‐cell response and significant tumor suppression. Combining the RP phage vaccine with ICIs offers a promising strategy for more effective cancer immunotherapy. The vaccine efficacy targeting the Adpgk neoantigen could further be enhanced by increasing the antigen density on the phage capsid, for example, by displaying more neoantigens on the pIII minor proteins. Overall, the RP phage‐based nanovaccine developed in this work demonstrated a strong anti‐tumor CD8^+^ T cell response, with uniform nanoparticle size and high stability, allowing for single‐step assembly at a potentially low production cost. These advancements pave the way for further research and optimization in personalized cancer vaccine development.

## 4 Experimental Section

### ssDNA Programming Algorithm and RP Phagemid

The ssDNA sequences of interest were generated using a custom‐developed algorithm based on a set of design principles. First, the flanking DNA sequence between the CG dimers was constrained to include the nucleotides ATCG, with the condition that it cannot start with C or end with G. Second, any repeating sequences of 9 bases or longer were addressed by mutating the A/T in the first instance of the repeating sequence to T/A, continuing this process until all repetitions were eliminated. Third, hairpin structures (defined as complementary DNA sequences longer than 12 bases) were removed by mutating A to T or T to A within these regions. Subsequently, gBlocks of the designed DNA sequences were synthesized by Integrated DNA Technologies.

To construct the RP phagemid, an inho phagemid was utilized.^[^ ^13^ ^]^ The RP phagemid features a ColE1 origin of replication and confers ampicillin resistance. It includes the f1 origin and terminator, along with a packaging signal, facilitating the production of high‐purity ssDNA segments with precise lengths. Specifically, the inho‐phagemid was linearized at the f1 origin and prior to the packaging signal using site‐directed mutagenesis, after which the gBlock of the designed DNA was inserted through Gibson assembly. The specific ssDNA sequences for RP phage CG0, CG09, CG27, and CpG40 can be found in Table S1 (Supporting Information).

### Helper Plasmid

The capsid plasmid features a p15a origin of replication and confers kanamycin resistance. It was derived from the RM13f1 helper plasmid of the inho‐phage system. To enable the display of the antigen of interest, a recombinant form of pVIII, regulated by the Tac promoter, was inserted between the M13 gene IV and the p15a origin of replication using site directed mutagenesis and Gibson assembly. The antigen sequence was incorporated at the N‐terminus of pVIII, either at the recombinant pVIII site or the wild‐type pVIII site, through site‐directed mutagenesis. The full pVIII sequences of the antigen pVIII‐expressing helper plasmids can be found in Table S3 (Supporting Information).

### Phage Amplification

Chemically competent XL1 blue cells (200249, Agilent) were co‐transformed with the helper plasmid and the RP phagemid, then plated on agar containing kanamycin (50 µg mL^−1^) and ampicillin (100 µg mL^−1^). A single colony was subsequently selected and cultured in 800 mL of LB broth supplemented with kanamycin (50 µg mL^−1^) and ampicillin (100 µg mL^−1^) for two consecutive overnight incubations at 37 °C with continuous shaking at 225 rpm. The bacterial cultures were then centrifuged at 8000 rpm (Beckman Coulter JLA 8.1000) for 1 h to pellet the bacterial cells. The supernatants containing phages were collected, and polyethylene glycol (PEG)/NaCl solution (final concentrations: 10% w/v PEG8000 and 0.5 M NaCl) was added to promote phage precipitation at 4 °C overnight. Following precipitation, the phage supernatants were centrifuged at 8000 rpm (Beckman Coulter JLA 8.1000) for 1 h to pellet the phage particles. The resulting phage pellets were resuspended in a solution of 1× TBS/MgCl_2_/DNase I (containing 5 µg mL^−1^ DNase I, 10 mM Tris‐HCl, and 10 mM MgCl_2_) and incubated for a minimum of 40 min at room temperature on a benchtop shaker to digest any DNA contaminants. After digestion, phage precipitation was facilitated by the addition of PEG/NaCl (10 w/v% PEG8000 and 0.5 M NaCl), and the mixture was incubated on ice for at least 2 h. The phage particles were then collected by centrifugation at 14 000 rpm for 10 min, and the phage pellets were resuspended in 1× PBS. Endotoxins were removed through a series of at least three washes with Triton 114. Specifically, Triton 114 (1 v/v%) was added to the phage solution, and the mixture was stirred at 4 °C for at least 1 h, followed by centrifugation at 14,000 rpm at 37 °C for a minimum of 30 min to remove the Triton 114. After centrifugation, the Triton 114 precipitated out of the phage solution, and the supernatant was carefully collected without disturbing the Triton 114 layer at the bottom. Following the final centrifugation, any remaining Triton 114 was removed using SM‐2 bio‐beads (1528920, Bio‐Rad Laboratories). The phage was then purified via CsCl gradient ultracentrifugation (1.2–1.6 g mL^−1^ gradient, SW32, 30,000 rpm, 4 °C overnight). After ultracentrifugation, the phage band, located at ≈1.3 g mL^−1^, was isolated and desalted through dialysis using a 10–12 kDa cut‐off membrane against Milli‐Q water for at least 24 h, with frequent buffer exchanges. Finally, the phage was concentrated again with PEG/NaCl (10 w/v% PEG and 0.5 M NaCl), centrifuged, and redispersed in 1× PBS for subsequent in vitro or in vivo applications.

### High Performance Liquid Chromatography

For antigen display density quantification with high performance liquid chromatography (HPLC), M13 phages were lysed with hot water (≈96 °C) assisted with sodium dodecyl sulfate (2 w/v%). HPLC was performed using an Agilent Model 1100 HPLC system equipped with an Agilent G1315A DAD Detector. Most of the chromatographic separation was achieved on a YMC‐Triart Bio C4 silica column (4.6 mm × 150 mm, 5 µm particle size, 300Å pore size) maintained at room temperature. The mobile phase consisted of 0.05% Trifluoroacetic Acid as Buffer A and 0.043% Trifluoroacetic acid, 80% Acetonitrile as Buffer B, delivered at a flow rate of 1.5 mL min^−1^. A gradient elution program was applied as follows: 0‐5 min isocratic at 5% Buffer B, 5‐75 min linear increase to 100% Buffer B. The injection volume was 95 µL (10 µL M13 phage + 90 µL 0.1% Trifluoroacetic acid, 5 µL dead volume).

### Matrix‐Assisted Laser Desorption/Ionization Time‐of‐Flight Mass Spectrometry

Matrix‐assisted laser desorption/ionization time‐of‐flight mass spectrometry (MALDI‐TOF MS) analyses were performed on a Bruker Microflex LRF with a N_2_ laser (337 nm) in positive ion mode. The mass spectrometer was operated in a linear mode with a mass range of 3000–8000 m/z and was calibrated to a 5729 Da standard. The laser energy was set at 60% of the maximum, and 400 shots per spectrum were averaged to improve the signal‐to‐noise ratio. The matrix used was sinapinic acid, prepared as a 20 mg mL^−1^ solution in 70% acetonitrile with 0.1% trifluoroacetic acid. The matrix solution was mixed with the sample in a 1:1 ratio, and 1 µL of this mixture was spotted onto the MALDI target plate using the dried droplet method. The target plate was allowed to dry at room temperature.

### In Vitro TLR9 Cell Assay

HEKblue mTLR9 (InvivoGen) reporter cells were employed to investigate TLR9 activation by the RP phages. Specifically, 4 × 10^4^ mTLR9 cells were seeded in a 96‐well plate overnight in 100 µL DMEM supplemented with 10% fetal bovine serum (FBS), 100 U mL^−1^ penicillin, and 100 U mL^−1^ streptomycin. 2 h prior to adding phage particles, the supernatant was replaced with DMEM medium supplemented with 10% heated‐inactivated FBS, 100 U mL^−1^ penicillin, and 100 U mL^−1^ streptomycin. RP phages (≈7.5 × 10^10^ phage particles calculated based on a ssDNA length of 7234 nucleotides) were then added to each well. To facilitate the delivery of the negatively charged ssDNA to the mTLR9 cells, the phage particles were heated‐inactivated by immersing them in water at 96 °C for 10 min and subsequently mixed with TransIT‐X2 (0.2 µL/well, Mirus Bio). Following overnight incubation, 90 µL of the supernatant was collected from each well and mixed with 90 µL of QUANTI‐Blue solution (rep‐qbs2, InvivoGen). The mixture was incubated at 37 °C for 15–30 min, and the absorbance at 650 nm was measured using a plate reader.

### Mice

All animal experiment procedures and protocols were pre‐approved by the Division of Comparative Medicine (DCM) and the Committee on Animal Care (CAC) under protocol #0222‐017‐25 at the Massachusetts Institute of Technology. These procedures complied with the Principles of Laboratory Animal Care established by the National Institutes of Health (NIH), United States.

All the mice used in the study were 8‐10 weeks‐old female C57/BL6J mice purchased from the Jackson Laboratory.

### Cell Lines and Cell Cultures

B16F10‐OVA cells (provided by the Irvine Lab, Koch Institute) and MC‐38 cells (obtained from the Koch Institute Cell Repository) were cultured in DMEM supplemented with 10% fetal bovine serum (FBS), 100 U mL^−1^ penicillin, and 100 U mL^−1^ streptomycin, and maintained at 37 °C in a 5% CO_2_ atmosphere.

### Lymph Node Draining and Intra‐Cellular Distribution Studies

Fluorescein (FAM)‐NHS ester (55120, Lumiprobe) was dissolved in anhydrous DMSO and reacted with the phage in 1× PBS overnight in the dark at room temperature. Following this reaction, excess FAM molecules were removed via dialysis (10–12 kDa cut‐off membrane) against 1× PBS for at least 24 h with frequent buffer exchange. The absorption spectrum of the resulting phage‐FAM complex was measured using a DU800 (Beckman) spectrophotometer.

For the lymph node drainage study, equivalent amounts of SIINFEKL peptide conjugated with FAM (SIINFEKL‐FAM, AS‐64231, Anaspec) and the phage‐FAM complex were prepared in 100 µL 1× PBS. Additionally, 100 µL 1× PBS was included as a control. These solutions were administered at the tail base of C57/BL6 mice. After 24 h, the inguinal, brachial, and axillary lymph nodes were excised intact, and their fluorescence (excitation: 465 nm; emission: 520 nm) was measured using an in vivo imaging system (IVIS).

Then the draining lymph nodes were dissociated into single cells and proceeded to antibody staining with the following makers: live/dead staining (L34976, Thermofisher), CD3e (BV786, clone 145‐2C11, 417‐0031‐82, eBioscience), B220/CD45R (PE, clone RA3‐6B2, 12‐0452‐82, eBioscience), CD11b (BV510, clone M1/70, 101263, Biolegend), CD11c (BV711, clone N418, 117349, Biolegend), F4/80 (APC, clone BM8, 123116, Biolegend), CD80 (PE‐Cy7, clone 16‐10A1, 104734, Biolegend) and CD86 (BV605, clone GL1, 105037, Biolegend).

### Immunohistology Staining of the Lymph Node

The draining lymph nodes were snap frozen with liquid nitrogen by embedding the tissue into the OCT compound in a Cryomold (Tissue‐Tek) and then cryosectioned. The tissue slices (10 µm thickness) were stained with CD3e (1:50, APC, clone 145‐2C11, 17‐0031‐82 eBioscience), B220 (1:50, PE, clone RA3‐6B2, 14‐0452‐82, eBioscience) and Hoechst 33342 dye (H1399, Thermofisher). The whole lymph node was scanned using the Leica SP8 Spectral Confocal Microscope. The images from individual channels were processed using Fiji software.

### In Vivo Immunization Studies

For the SIINFEKL vaccination studies, two doses of RP phages displaying the SIINFEKL pVIIIs (5 × 10^12^ phage particles calculated based on a ssDNA length of 7234 nucleotides, 100 µL 1× PBS), or an equivalent amount of free SIINFEKL peptide (≈3.8 nmol, ≈3.7 µg) mixed with CpG oligonucleotide (≈3.6 nmol, 5’‐ TCCATGACGTTCCTGACGTT‐3’, Integrated DNA Technologies) were administrated via tail base injection on day 0 and day 7. For Fig 5b, the RP phage dose was 3.3×10^12^ phage particles. On day 14, ≈30 µL of blood was used to study the antigen‐specific CD8^+^ T cell response. Specifically, red blood cells (RBCs) were lysed using 300 µL of RBC lysis buffer (R7757, Millipore Sigma). The peripheral blood mononuclear cells (PBMCs) were then collected by centrifugation at 500 g for 5 min and the supernatant was discarded. An additional 300 µL of RBC lysis buffer was added to further lyse any remaining RBCs, followed by a second centrifugation at 500 g for 5 min. After discarding the supernatant, the PBMCs were redispersed in 100 µL 1× PBS containing LIVE/DEAD fixable near‐IR dye (L34976, Themofisher) for live/dead staining at room temperature for 10 min. The cells were centrifuged at 500 g for 5 min and then redispersed in 100 µL FACS buffer (1% BSA in 1× PBS) containing Fc blocker (101320, Biolegend), SIINFEKL H‐2K^b^ tetramer (PE, TB‐5001‐1, MBL international) and anti‐mouse CD8α antibody (APC, GTX76346, GeneTex) at room temperature for 30 min. Excess antibodies were removed through two centrifugation steps at 500 g for 5 min each.

For the Adpgk neoantigen in vivo vaccination study, RP phages displaying the Adpgk pVIIIs (5×10^12^ phage particles, calculated based on a ssDNA length of 7234 nucleotides, 100 µL 1× PBS), or equivalent amount of free Adpgk peptide (≈15.8 µg, Elim Biopharmaceuticals) mixed with CpG oligonucleotide (≈3.6 nmol) were administrated via tailbase injection. The H‐2D^b^ Adpgk neoepitope Tetramer (PE, TB‐5113‐1, MBL International) and the anti‐mouse CD279 (PD‐1) Antibody (BV421, clone 29F1A12, 135221, BioLegend) were used for the cell staining.

For prophylactic tumor challenge studies, on day 15, B16F10‐OVA cells or MC‐38 cells in 100 µL 1× PBS were subcutaneously inoculated at the left flank of the mice. Tumor size was monitored every 2‐4 days and calculated using the empirical formula: $V=\frac{1}{2}\times \mathrm{length}\times {\mathrm{width}}^{2}$. Mice were euthanized if the tumor volume exceeded 1000 mm^3^ or when animals became moribund with severe weight loss or ulceration.

### Cancer Immunotherapy Studies

For the MC‐38 colon cancer treatment study, MC‐38 colon cancer was first established by subcutaneous injection of 0.25 million cells (in 100 µL 1× PBS) in the left flank of the mouse. 8 days post tumor inoculation when the average tumor size reached ≈80 mm^3^, the mice were regrouped to make sure each study group had similar size distributions. RP phages (1.0×10^13^ phage particles, calculated based on a ssDNA length of 7234 nucleotides, 100 µL 1× PBS) or equivalent combinations of free Adpgk peptide (≈31.6 µg) and CpG (≈7.2 nmol) were administered in two doses on day 8 and 15 post tumor inoculation. Anti‐mouse PD‐1 antibodies (100 µg, clone RMP1‐14, BioXcell, 1× PBS) were administered on day 2 and day 4 following each vaccination treatment, or alone at the same time. Tumor size was monitored every 2‐4 days and calculated using the empirical formula: $V=\frac{1}{2}\times \mathrm{length}\times \mathrm{widt}h^{2}$. Mouse were euthanized if the tumor volume exceeded 1000 mm^3^ or when animals became moribund with severe weight loss or ulceration.

## Conflict of Interest

The authors declare no conflict of interest.

## Author Contributions

S.H. and A.M.B conceived the idea and designed the experiments. S.H. constructed the plasmids with help from Y.H. and N.H. S.H. and Y.H. contributed to the tetramer staining assays. S.H., A.M., and M.G. purified the phages and maintained the cell culture. S.H., A.M., and H.P. conducted the lymph node draining experiments. H.P. carried out the immunohistology staining and fluorescence microscopy. M.H. and S.H. developed the algorithms for programming the ssDNA and fitting the HPLC peaks. J.Q. contributed to the TEM images. H.A. and R.A. performed the HPLC and MALDI‐TOF MS. S.H. and A.M.B analyzed the results and wrote the manuscript. All the authors reviewed the manuscript.

## Supporting information



Supporting Information

## Data Availability

The data that support the findings of this study are available from the corresponding author upon reasonable request.

## References

[1] H. Qin , R. Zhao , Y. Qin , J. Zhu , L. Chen , C. Di , X. Han , K. Cheng , Y. Zhang , Y. Zhao , J. Shi , G. J. Anderson , Y. Zhao , G. Nie , Adv. Mater. 2021, 33, 2006007. 10.1002/adma.202006007 33792097

[2] R. Kuai , L. J. Ochyl , K. S. Bahjat , A. Schwendeman , J. J. Moon , Nature Mater 2017, 16, 489. 28024156 10.1038/nmat4822 PMC5374005

[3] F. Shen , H. Wang , Z. Liu , L. Sun , Angew Chem Int Ed 2024, 63, 202312624. 10.1002/anie.202312624 37737971

[4] P. Huang , L. Jiang , H. Pan , L. Ding , B. Zhou , M. Zhao , J. Zou , B. Li , M. Qi , H. Deng , Y. Zhou , X. Chen , Adv. Mater. 2023, 35, 2207471. 10.1002/adma.202207471 36326183

[5] J. Nam , S. Son , K. S. Park , J. J. Moon , Adv. Sci. 2021, 8, 2002577. 10.1002/advs.202002577 PMC7927624 33717838

[6] H. Yue , Y. Li , T. Yang , Y. Wang , Q. Bao , Y. Xu , X. Liu , Y. Miao , M. Yang , C. Mao , Nat. Nanotechnol. 2025, 20, 167. 39468354 10.1038/s41565-024-01800-4

[7] S. Huang , J. Qi , D. W. deQuilettes , M. Huang , C. Lin , N. M. Bardhan , X. Dang , V. Bulović , A. M. Belcher , Small 2019, 15, 1901233. 10.1002/smll.201901233 31131998

[8] D. I. Staquicini , F. H. F. Tang , C. Markosian , V. J. Yao , F. I. Staquicini , E. Dodero‐Rojas , V. G. Contessoto , D. Davis , P. O'Brien , N. Habib , T. L. Smith , N. Bruiners , R. L. Sidman , M. L. Gennaro , E. C. Lattime , S. K. Libutti , P. C. Whitford , S. K. Burley , J. N. Onuchic , W. Arap , R. Pasqualini , Proc. Natl. Acad. Sci. U.S.A. 2021, 118, 2105739118. 10.1073/pnas.2105739118 PMC8325333 34234013

[9] S.‐W. Lee , C. Mao , C. E. Flynn , A. M. Belcher , Science 2002, 296, 892. 11988570 10.1126/science.1068054

[10] C. Chang , W. Guo , X. Yu , C. Guo , N. Zhou , X. Guo , R.‐L. Huang , Q. Li , Y. Zhu , Materials Today Bio 2023, 20, 100612. 10.1016/j.mtbio.2023.100612 PMC10102448 37063776

[11] R. M. Dedrick , C. A. Guerrero‐Bustamante , R. A. Garlena , D. A. Russell , K. Ford , K. Harris , K. C. Gilmour , J. Soothill , D. Jacobs‐Sera , R. T. Schooley , G. F. Hatfull , H. Spencer , Nat. Med. 2019, 25, 730. 31068712 10.1038/s41591-019-0437-z PMC6557439

[12] A. Petrovic Fabijan , R. C. Y. Lin , J. Ho , S. Maddocks , N. L. Ben Zakour , J. R. Iredell , Westmead Bacteriophage Therapy Team , A. Khalid , C. Venturini , R. Chard , S. Morales , I. Sandaradura , T. Gilbey , Nat. Microbiol. 2020, 5, 465. 32066959 10.1038/s41564-019-0634-z

[13] U. Tsedev , C.‐W. Lin , G. T. Hess , J. N. Sarkaria , F. C. Lam , A. M. Belcher , ACS Nano 2022, 16, 11676. 35830573 10.1021/acsnano.1c08720

[14] T. Yang , Q. Zhang , Y. Miao , Y. Lyu , Y. Xu , M. Yang , C. Mao , Adv. Mater. 2025, 37, 2403756. 39233557 10.1002/adma.202403756 PMC11733710

[15] G. P. Smith , Angew Chem Int Ed 2019, 58, 14428.

[16] Y. Huai , S. Dong , Y. Zhu , X. Li , B. Cao , X. Gao , M. Yang , L. Wang , C. Mao , Adv Healthcare Materials 2016, 5, 786. 10.1002/adhm.201500930 PMC4828319 26890982

[17] Y. Wang , G. Zhang , L. Zhong , M. Qian , M. Wang , R. Cui , Nanoscale 2022, 14, 5942. 35389413 10.1039/d1nr08064d

[18] Q. Bao , X. Li , G. Han , Y. Zhu , C. Mao , M. Yang , Adv. Drug Delivery Rev. 2019, 145, 40. 10.1016/j.addr.2018.12.013 30594492

[19] S. Hashiguchi , Y. Yamaguchi , O. Takeuchi , S. Akira , K. Sugimura , Biochem. Biophys. Res. Commun. 2010, 402, 19. 20875795 10.1016/j.bbrc.2010.09.094

[20] L. L. Cao , J. C. Kagan , Immunity 2023, 56, 2206. 37703879 10.1016/j.immuni.2023.07.018 PMC10591974

[21] X. Dong , P. Pan , D.‐W. Zheng , P. Bao , X. Zeng , X.‐Z. Zhang , Sci. Adv. 2020, 6, aba1590. 10.1126/sciadv.aba1590 PMC7228756 32440552

[22] L. Lei , J. Yan , K. Xin , L. Li , Q. Sun , Y. Wang , T. Chen , S. Wu , J. Shao , B. Liu , X. Chen , ACS Nano 2024, 18, 12194. 38689426 10.1021/acsnano.4c00413

[23] H. Shi , S. Dong , X. Zhang , X. Chen , X. Gao , L. Wang , Vaccine 2018, 36, 5717. 30111514 10.1016/j.vaccine.2018.08.011

[24] D. Frenkel , O. Katz , B. Solomon , Proc. Natl. Acad. Sci. U.S.A. 2000, 97, 11455. 11027345 10.1073/pnas.97.21.11455 PMC17221

[25] C. Bartolacci , C. Andreani , C. Curcio , S. Occhipinti , L. Massaccesi , M. Giovarelli , R. Galeazzi , M. Iezzi , M. Tilio , V. Gambini , J. Wang , C. Marchini , A. Amici , Cancer Immunol. Res. 2018, 6, 1486. 30327365 10.1158/2326-6066.CIR-18-0179

[26] V. Lavie , M. Becker , R. Cohen‐Kupiec , I. Yacoby , R. Koppel , M. Wedenig , B. Hutter‐Paier , B. Solomon , JMN 2004, 24, 105. 10.1385/JMN:24:1:105 15314258

[27] R. Sartorius , L. D'Apice , P. Barba , D. Cipria , L. Grauso , A. Cutignano , P. De Berardinis , Front. Immunol. 2018, 9, 1496. 30002659 10.3389/fimmu.2018.01496 PMC6031736

[28] L. Van Oosten , J. J. Altenburg , C. Fougeroux , C. Geertsema , F. Van Den End , W. A. C. Evers , A. H. Westphal , S. Lindhoud , W. Van Den Berg , D. C. Swarts , L. Deurhof , A. Suhrbier , T. T. Le , S. Torres Morales , S. K. Myeni , M. Kikkert , A. F. Sander , W. A. De Jongh , R. Dagil , M. A. Nielsen , A. Salanti , M. Søgaard , T. M. P. Keijzer , D. Weijers , M. H. M. Eppink , R. H. Wijffels , M. M. Van Oers , D. E. Martens , G. P. Pijlman , mBio 2021, 12, 01813. 10.1128/mBio.01813-21 PMC8510518 34634927

[29] H. Liu , H. Chen , Z. Yang , Z. Wen , Z. Gao , Z. Liu , L. Liu , Y. Chen , JACS Au 2024, 4, 2792. 39211600 10.1021/jacsau.4c00568 PMC11350730

[30] R. A. Gottschalk , M. M. Hathorn , H. Beuneu , E. Corse , M. L. Dustin , G. Altan‐Bonnet , J. P. Allison , Proc. Natl. Acad. Sci. U.S.A. 2012, 109, 881. 22223661 10.1073/pnas.1119763109 PMC3271915

[31] S. G. Reed , M. T. Orr , C. B. Fox , Nat. Med. 2013, 19, 1597. 24309663 10.1038/nm.3409

[32] A. Dalpke , J. Frank , M. Peter , K. Heeg , Infect. Immun. 2006, 74, 940. 16428738 10.1128/IAI.74.2.940-946.2006 PMC1360326

[33] A. M. Krieg , A.‐K. Yi , S. Matson , T. J. Waldschmidt , G. A. Bishop , R. Teasdale , G. A. Koretzky , D. M. Klinman , Nature 1995, 374, 546. 7700380 10.1038/374546a0

[34] C.‐Y. Lai , G.‐Y. Yu , Y. Luo , R. Xiang , T.‐H. Chuang , Front. Immunol. 2019, 10, 179. 30800129 10.3389/fimmu.2019.00179 PMC6375897

[35] H. G. Rammensee , K. Falk , O. Rötzschke , Annu. Rev. Immunol. 1993, 11, 213. 8476560 10.1146/annurev.iy.11.040193.001241

[36] R. Sartorius , C. Bettua , L. D'Apice , A. Caivano , M. Trovato , D. Russo , I. Zanoni , F. Granucci , D. Mascolo , P. Barba , G. Del Pozzo , P. De Berardinis , Eur. J. Immunol. 2011, 41, 2573. 21688262 10.1002/eji.201141526

[37] P. Malik , R. N. Perham , Nucleic Acids Res. 1997, 25, 915. 9016648 10.1093/nar/25.4.915 PMC146502

[38] Y. Sykulev , M. Joo , I. Vturina , T. J. Tsomides , H. N. Eisen , Immunity 1996, 4, 565. 8673703 10.1016/s1074-7613(00)80483-5

[39] S. W. Smeal , M. A. Schmitt , R. R. Pereira , A. Prasad , J. D. Fisk , Virology 2017, 500, 259. 27644585 10.1016/j.virol.2016.08.017

[40] R. Conners , R. I. León‐Quezada , M. McLaren , N. J. Bennett , B. Daum , J. Rakonjac , V. A. M. Gold , Nat. Commun. 2023, 14, 2724. 37169795 10.1038/s41467-023-37915-w PMC10175506

[41] R. Conners , M. McLaren , U. Łapińska , K. Sanders , M. R. L. Stone , M. A. T. Blaskovich , S. Pagliara , B. Daum , J. Rakonjac , V. A. M. Gold , Nat. Commun. 2021, 12, 6316. 34728631 10.1038/s41467-021-26610-3 PMC8563730

[42] G. T. Hess , J. J. Cragnolini , M. W. Popp , M. A. Allen , S. K. Dougan , E. Spooner , H. L. Ploegh , A. M. Belcher , C. P. Guimaraes , Bioconjugate Chem. 2012, 23, 1478. 10.1021/bc300130z PMC3564602 22759232

[43] H. Liu , K. D. Moynihan , Y. Zheng , G. L. Szeto , A. V. Li , B. Huang , D. S. Van Egeren , C. Park , D. J. Irvine , Nature 2014, 507, 519. 24531764 10.1038/nature12978 PMC4069155

[44] M. F. Bachmann , G. T. Jennings , Nat. Rev. Immunol. 2010, 10, 787. 20948547 10.1038/nri2868

[45] X. Yu , Y. Dai , Y. Zhao , S. Qi , L. Liu , L. Lu , Q. Luo , Z. Zhang , Nat. Commun. 2020, 11, 1110. 32111828 10.1038/s41467-020-14906-9 PMC7048802

[46] G. Zhu , L. Mei , H. D. Vishwasrao , O. Jacobson , Z. Wang , Y. Liu , B. C. Yung , X. Fu , A. Jin , G. Niu , Q. Wang , F. Zhang , H. Shroff , X. Chen , Nat. Commun. 2017, 8, 1482. 29133898 10.1038/s41467-017-01386-7 PMC5684198

[47] Q. Ni , F. Zhang , Y. Liu , Z. Wang , G. Yu , B. Liang , G. Niu , T. Su , G. Zhu , G. Lu , L. Zhang , X. Chen , Sci. Adv. 2020, 6, aaw6071. 10.1126/sciadv.aaw6071 PMC7080439 32206706

[48] N. Brooks , S. Esparon , D. Pouniotis , G. Pietersz , Molecules 2015, 20, 14033. 26247926 10.3390/molecules200814033 PMC6332296

[49] J. Deeg , M. Axmann , J. Matic , A. Liapis , D. Depoil , J. Afrose , S. Curado , M. L. Dustin , J. P. Spatz , Nano Lett. 2013, 13, 5619. 24117051 10.1021/nl403266t PMC3828117

[50] R. Billeskov , B. Beikzadeh , J. A. Berzofsky , Hum. Vaccines Immunother. 2019, 15, 407. 10.1080/21645515.2018.1527496 PMC6422501 30277831

[51] S. L. Demento , W. Cui , J. M. Criscione , E. Stern , J. Tulipan , S. M. Kaech , T. M. Fahmy , Biomaterials 2012, 33, 4957. 22484047 10.1016/j.biomaterials.2012.03.041 PMC5724530

[52] S. M. Kaech , E. J. Wherry , R. Ahmed , Nat. Rev. Immunol. 2002, 2, 251. 12001996 10.1038/nri778

[53] K. D. Omilusik , A. W. Goldrath , Curr. Opin. Immunol. 2019, 58, 89. 31170601 10.1016/j.coi.2019.04.009 PMC6612439

[54] S. M. Alam , P. J. Travers , J. L. Wung , W. Nasholds , S. Redpath , S. C. Jamesont , N. R. J. Gascoigne , Nature 1996, 381, 616. 8637599 10.1038/381616a0

[55] S. M. Alam , G. M. Davies , C. M. Lin , T. Zal , W. Nasholds , S. C. Jameson , K. A. Hogquist , N. R. J. Gascoigne , P. J. Travers , Immunity 1999, 10, 227. 10072075 10.1016/s1074-7613(00)80023-0

[56] D. K. Cole , N. J. Pumphrey , J. M. Boulter , M. Sami , J. I. Bell , E. Gostick , D. A. Price , G. F. Gao , A. K. Sewell , B. K. Jakobsen , J. Immunol. 2007, 178, 5727. 17442956 10.4049/jimmunol.178.9.5727

[57] B. A. Kansy , F. Concha‐Benavente , R. M. Srivastava , H.‐B. Jie , G. Shayan , Y. Lei , J. Moskovitz , J. Moy , J. Li , S. Brandau , S. Lang , N. C. Schmitt , G. J. Freeman , W. E. Gooding , D. A. Clump , R. L. Ferris , Cancer Res. 2017, 77, 6353. 28904066 10.1158/0008-5472.CAN-16-3167 PMC5690836

[58] H. Arasanz , M. Gato‐Cañas , M. Zuazo , M. Ibañez‐Vea , K. Breckpot , G. Kochan , D. Escors , Oncotarget 2017, 8, 51936. 28881701 10.18632/oncotarget.17232 PMC5584302

[59] E. Ahn , K. Araki , M. Hashimoto , W. Li , J. L. Riley , J. Cheung , A. H. Sharpe , G. J. Freeman , B. A. Irving , R. Ahmed , Proc. Natl. Acad. Sci. U.S.A. 2018, 115, 4749. 29654146 10.1073/pnas.1718217115 PMC5939075

[60] S. Simon , N. Labarriere , OncoImmunology 2018, 7, 1364828. 10.1080/2162402X.2017.1364828 PMC5739549 29296515

[61] Y. He , C. Hong , S. Huang , J. A. Kaskow , G. Covarrubias , I. S. Pires , J. C. Sacane , P. T. Hammond , A. M. Belcher , Adv Healthcare Materials 2023, 12, 2300688. 10.1002/adhm.202300688 PMC10964211 37015729

[62] W. Zou , J. D. Wolchok , L. Chen , Sci. Transl. Med. 2016, 8, 328rv4. 10.1126/scitranslmed.aad7118 PMC4859220 26936508

[63] S. Huang , C. Lin , J. Qi , A. M. Iyer , Y. He , Y. Li , N. M. Bardhan , D. J. Irvine , P. T. Hammond , A. M. Belcher , Adv. Mater. 2021, 33, 2006057. 10.1002/adma.202006057 33448062

[64] A. M. D'Alise , N. Brasu , C. De Intinis , G. Leoni , V. Russo , F. Langone , D. Baev , E. Micarelli , L. Petiti , S. Picelli , M. Fakih , D. T. Le , M. J. Overman , A. F. Shields , K. S. Pedersen , M. A. Shah , S. Mukherjee , T. Faivre , P. Delaite , E. Scarselli , L. Pace , Sci. Transl. Med. 2022, 14, abo7604. 10.1126/scitranslmed.abo7604 PMC9844517 35947675

[65] T. Ozasa , M. Nakajima , R. Tsunedomi , S. Goto , K. Adachi , H. Takahashi , K. Tamada , H. Nagano , Sci. Rep. 2025, 15, 8956. 40089538 10.1038/s41598-025-87344-6 PMC11910518

[66] L. Liu , J. Chen , H. Zhang , J. Ye , C. Moore , C. Lu , Y. Fang , Y.‐X. Fu , B. Li , Nat Cancer 2022, 3, 437. 35393580 10.1038/s43018-022-00352-7 PMC9050907

[67] Q. Yin , L. Wu , L. Han , X. Zheng , R. Tong , L. Li , L. Bai , Y. Bian , Front. Immunol. 2023, 14, 1167975. 37304306 10.3389/fimmu.2023.1167975 PMC10247998

[68] B. Reynisson , B. Alvarez , S. Paul , B. Peters , M. Nielsen , Nucleic Acids Res. 2020, 48, W449. 32406916 10.1093/nar/gkaa379 PMC7319546

[69] G. Li , B. Iyer , V. B. S. Prasath , Y. Ni , N. Salomonis , Briefings in Bioinformatics 2021, 22, bbab160. 34009266 10.1093/bib/bbab160 PMC8135853

