# Supplementary material for: A Programmable Nanovaccine Platform Based on M13 Bacteriophage for Personalized Cancer Vaccine and Therapy
Source: Adv Mater. 2025 Aug 27;37(43):e10229. doi: 10.1002/adma.202510229 (PMC12574644; doi:10.1002/adma.202510229)
Supplement: Supplementary file 1 — Supporting Information [file ADMA-37-e10229-s001.pdf]

ADVANCED MATERIALS

Supporting Information

for *Adv. Mater.*, DOI 10.1002/adma.202510229

A Programmable Nanovaccine Platform Based on M13 Bacteriophage for Personalized Cancer Vaccine and Therapy

*Shengnan Huang, Yanpu He, Allison Madow, Huaiyao Peng, Mirielle Griffin, Jifa Qi, Mantao Huang, Heather Amoroso, Riley Abrashoff, Nimrod Heldman and Angela M. Belcher**

Supporting Information for

A programmable nanovaccine platform based on M13 bacteriophage for personalized cancer vaccine and therapy

Shengnan Huang[1], Yanpu He[1], Allison Madow[1], Huaiyao Peng[1,2], Mirielle Griffin[3], Jifa Qi[1], Mantao Huang[4], Heather Amoroso[5], Riley Abrashoff[5], Nimrod Heldman[1], Angela M. Belcher[1,2,6]*

1. The David H. Koch Institute for Integrative Cancer Research, Massachusetts Institute of Technology; Cambridge, MA, 02139, USA.

2. Department of Biological Engineering, Massachusetts Institute of Technology; Cambridge, MA, 02139, USA.

3. Department of Brain and Cognitive Sciences, Massachusetts Institute of Technology; Cambridge, MA, 02139, USA.

4. Department of Nuclear Science and Engineering, Massachusetts Institute of Technology; Cambridge, MA, 02139, USA.

5. Biopolymers Core Lab, The David H. Koch Institute for Integrative Cancer Research, Massachusetts Institute of Technology; Cambridge, MA, 02139, USA.

6. Department of Materials Science and Engineering, Massachusetts Institute of Technology; Cambridge, MA, 02139, USA.

* email: belcher@mit.edu

This PDF file includes:

Figs. S1 to S16

Tables S1 to S3

Fig. S1. Dose study of the HEKBlue mTLR9 activation by the reprogrammed ssDNAs

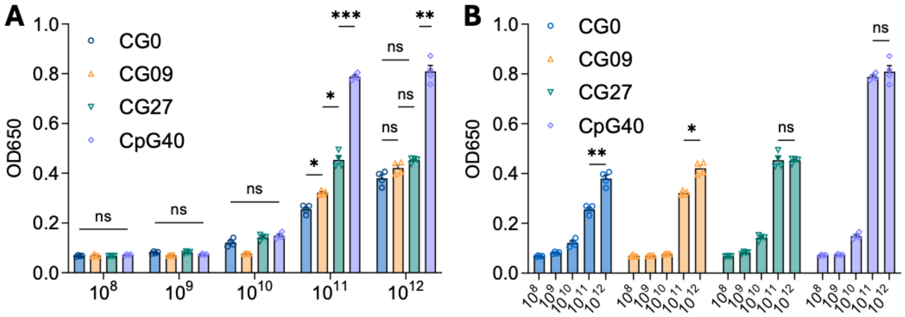

Fig. S1. Dose study of the HEKBlue mTLR9 activation with various ssDNAs. (**A**) Grouped by the dose and (**B**) grouped by the ssDNA. The data show mean ± s.e.m.. ns, not significant, ∗$p<0.05$, ∗∗$p<0.01$, and ∗∗∗$p<0.001$, analyzed by two-way ANOVA(**A**, **B**) with Bonferroni post hoc test.

The HEKBlue mTLR9 cells were seeded in a 96-well plate overnight. Then 10^8–10^{12} phages (calculated based on ssDNA base of 7234) were added to each well (see methods for the details). After 24 hours of incubation, the secreted embryonic alkaline phosphatase (SEAP) in the cell supernatant was measured. There was little mTLR9 activation for phage dose below 10^{10}/well and the activation of CG27 and CpG40 saturated above 10^{11}/well. Hence, a phage dose above 10^{10}/well and below 10^{11}/well was suitable to study the mTLR9 activation of different ssDNAs and the dose we used in our study was ~ 7.5×10^{10}/well.

Our dosage study indicated among all the different ssDNAs (CG0,09,CG27 and CpG40), CpG40 generated the highest SEAP among all the phage doses (10^8–10^{12}/well). Particularly, when CG27 reached the SEAP production plateau at the highest phage dose of 10^{11} and 10^{12}/well, the CpG40 also reached the SEAP production plateau, however, with higher NF-κB activation and SEAP production. This suggested that the canonical CpG hexamers (GACGTT, AACGTT) in the CpG40 demonstrated better NF-κB activation than the CG dimers, as the canonical CpG hexamers exhibit stronger binding towards the TLR9 receptor, promoting more efficient TLR9 receptor dimerization and downstream NF-κB activation.

Fig. S2. SIINFEKL pVIII characterization with HPLC and MALDI-TOF mass spectrometry

SIINFEKL pVIII-expressing RP phages were amplified with an RP phage phagemid and the rEES helper plasmid. Various fractions (fxns) were collected in Fig. S2A at the characteristic peaks as indicated by different colors and analyzed with MALDI-TOF MS (Fig. S2B). The main peak at ~ 55.8 min (fxn 56 and part of fxn 57) was identified to be the wild-type pVIII with molecular weight of 5329 Da and the shoulder peak following it at ~ 56.5min (part of fxn 57) was the SIINFEKL pVIII with molecular weight of 6142 Da.

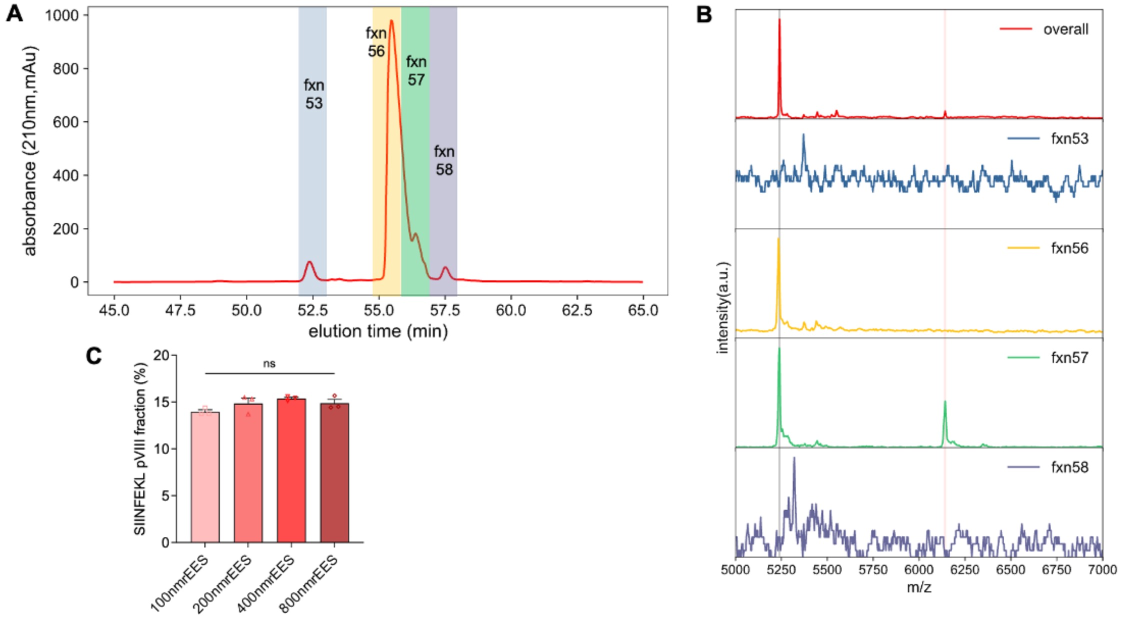

Fig. S2. Identification of SIINFEKL pVIII with HPLC and MALDI-TOF MS. (**A**) Various factions of the SIINFEKL pVIII-displaying phages were collected with HPLC as indicated by different colors. (**B**) MALDI-TOF MS plot of the various HPLC fractions collected in (**A**). (**C**) The SIINFEKL pVIII display ratio was independent of the phage length. The data show mean ± s.e.m.. ns, not significant, analyzed by one-way ANOVA (**C**) with Bonferroni post hoc test.

Fig. S3. FAM conjugation and quantification on the RP phages

The fluorescein (FAM) density on the phage surface (number of FAM molecules per phage particle) was quantified by using absorption measurement. First, a linear calibration curve of absorbance vs. dye concentration was constructed by measuring the absorbance of the free FAM at various concentrations as in Fig. S3A and B. To characterize the FAM density on the phage surface, the absorption spectrum of the phage-FAM was measured making sure that the peak absorbance fell in the linear absorption regime (Fig. S3C as the normalized absorption spectrum of the phage-FAM complex). Then the FAM concentration was determined from the calibration curve and the FAM density on the phage surface was determined as $[FAM](M)/[phage](M)$. For a typical phage-FAM complexation study, on average there were ~ 680 FAM molecules conjugated on each phage (the phage particle concentration was calculated based on the ssDNA length of 7234 nucleotides).

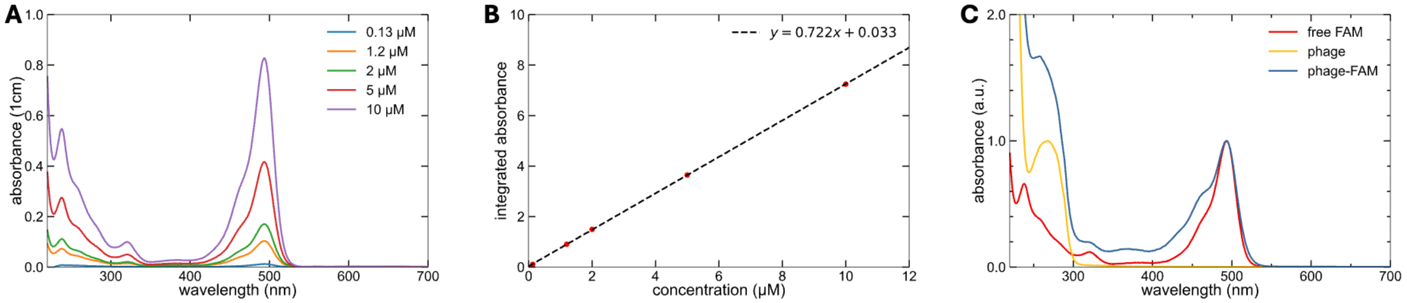

Fig. S3. **Quantification of the FAM density on the RP phage with absorption measurement.** (**A**) Absorption spectrum of free FAM in 1× PBS at various concentrations. (**B**) Linear calibration curve of the absorbance vs. concentration for the free FAM. (**C**) Normalized absorption spectrum of phage, free FAM and phage-FAM complex confirmed the conjugation of FAM on the phage surface.

Fig. S4. FACS gating of the phage distribution among immune cells and activation of immune cells in draining lymph nodes

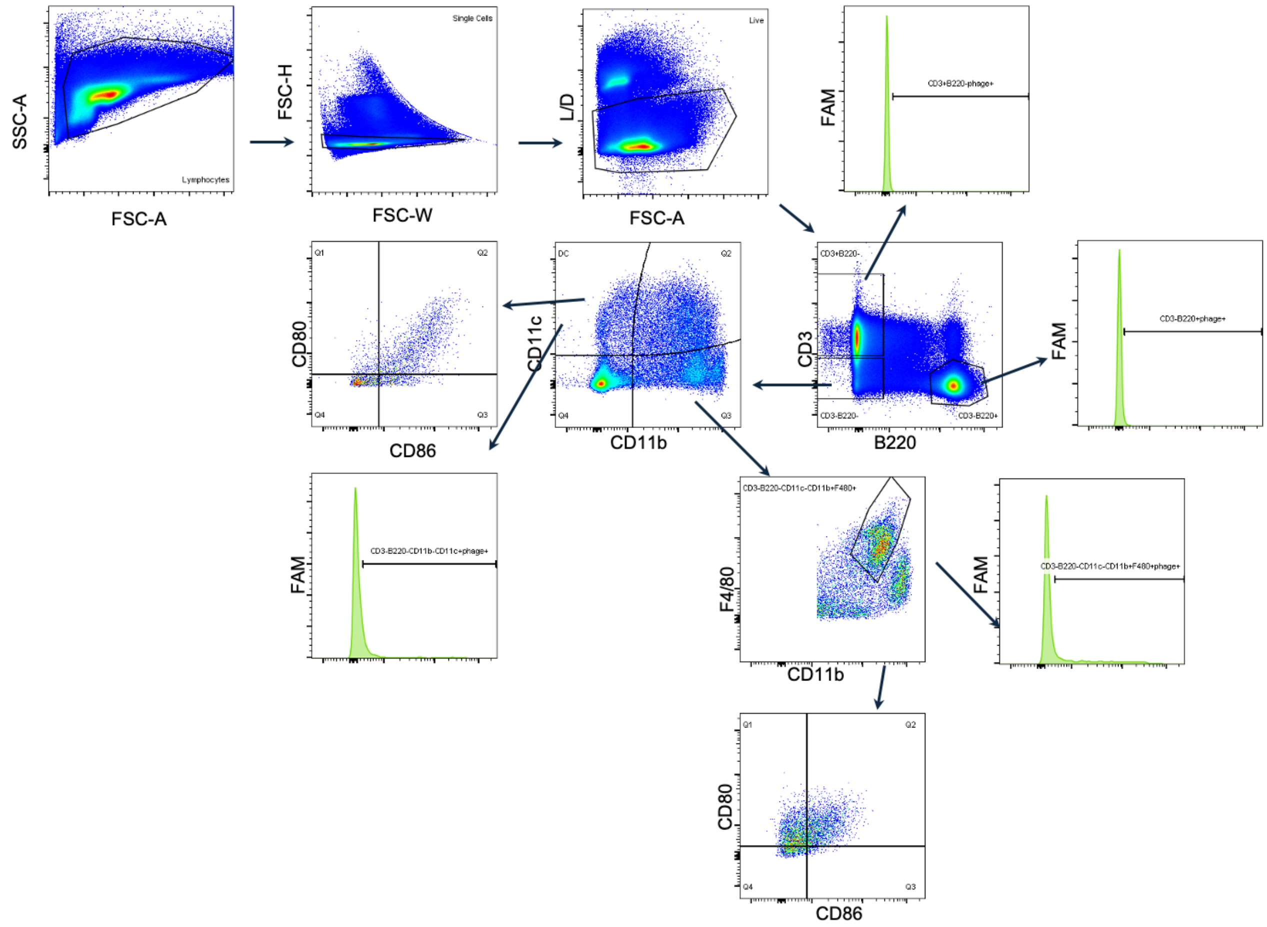

Fig. S5. Lower antigen display at constant adjuvanticity decreased the antigen-specific CD8+ T cell response

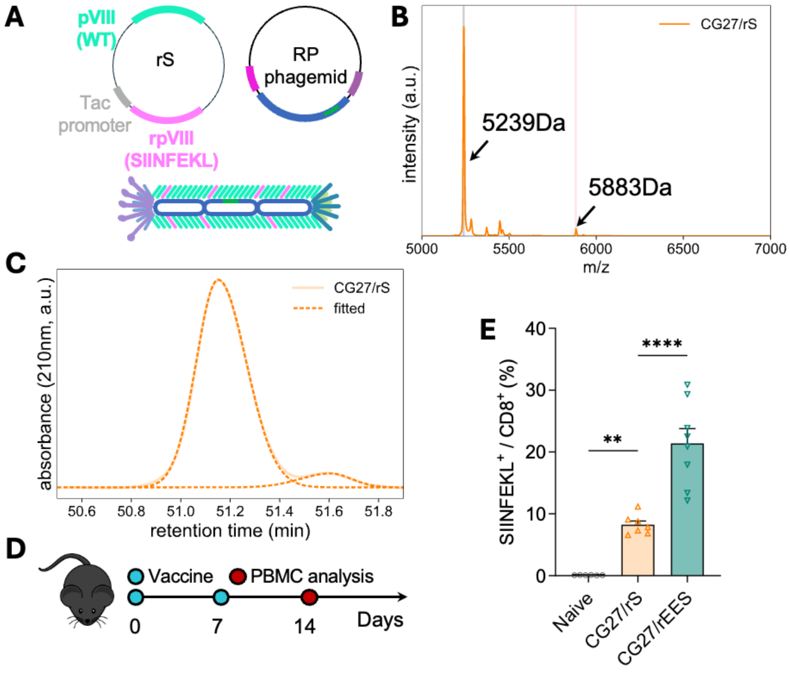

Fig. S5. Decreasing the antigen display below 13.6% at constant adjuvanticity (CG27) resulted in lower antigen-specific CD8$^+$ T cell response. (**A**) Amplification of RP phages combining the helper plasmid rS and the RP phagemid. (**B**) MALDI-TOF confirmed the antigen pVIII at 5883Da. (**C**) HPLC measurement showed ~ 6% of the antigen pVIII display ratio. (**D**) Vaccination schedule. 5×10^{12} phage particles (calculated based on ssDNA base of 7234) were administered subcutaneously at tail base on day 0 and 7. PBMCs were analyzed for SIINFEKL-specific CD8$^+$ T cell response with flow cytometry. (**E**) Decreasing the antigen density resulted in lower antigen-specific CD8$^+$ T cell frequency. The data show mean ± s.e.m.. **p<0.01 and ***p<0.001, analyzed by one-way ANOVA (**E**) with Bonferroni post hoc test.

In order to study the antigen density effect on anti-tumor CD8$^+$ T cell response, we further decreased the antigen pVIII display ratio below 13.6% with a new helper plasmid rS, in which the antigen peptide sequence of SIINFEKL was displayed at the N-terminus of the recombinant pVIII under the control of a Tac promoter (Fig. S5A). MALDI-TOF confirmed the coexistence of the wild type pVIII (5239Da) and the antigen pVIII (5883Da) (Fig. S5B), and HPLC measured the antigen pVIII display ration to be ~ 6% (Fig. S5C). The decrease in the antigen pVIII display by the rS helper plasmid compared to the rEES plasmid could be due to the charge differences associate with the antigen peptide SIINFEKL and EESIINFEKL. As the N-terminus of pVIII is exposed to the solvent, thus negatively charged hydrophilic amino acid residues was preferred for N-terminus display. The peptide SIINFEKL has less negatively charged amino acid residues and may not be favorable for high density display on pVIIIs. However, the exact mechanism needs further study.

In combination with the CG27 phagemid, the CG27/rS phages were examined in vivo for the vaccine efficacy following the dosing schedule in Fig. S5D, demonstrating that the SIINFEKL-specific CD8$^+$ T cell frequency increased from 8.2% to 21.4% (Fig. S5E), when increasing the antigen density from 6% to 13.6%. Hence, the antigen density plays a critical role in determining the anti-tumor CD8$^+$ T cell response.

Fig. S6. RP phages of various length and adjuvanticity exhibit similar zeta potential

Zeta potentials of the phages of varying adjuvanticity (CG0,09,27,CpG40/rEES) and different length (100nm, 200nm, 400nm, 800nm/rEES) were measured to study their stability.

All the phages of varying adjuvanticity but similar length of 200 nm, exhibited zeta potential of ~–25mV (Fig. S6A), indicating that the zeta potential depend little on the adjuvanticity of the phage. Particularly, all the phages were stored at 4 ºC for about 18 months when conducting the measurement and they showed little variation from the newly amplified phage (CG27/rEES*, stored at 4 ºC for about 3 months), demonstrating their superior stability.

In addition, our results also demonstrated that the zeta potential depended little on the length of the phage, with ~–25mV for the 100 nm phages and ~–30mV for the 800 nm phages (Fig S6B). The slightly more negative zeta potential of longer phages could be explained by the cylindrical shape of the phage, with minor proteins at the two ends and major coat proteins along the cylinder. Since the major coat proteins contribute primarily to the surface charge of the M13 phages, shorter phages with slightly higher percentage of minor coat proteins than longer phages exhibit a little more positive zeta potential. However, all the phages of various lengths demonstrate superior stability over time (Fig. S6B, old: stored at 4 ºC for about 18 months, new: stored at 4 ºC for about 3 months). Overall, the results indicate that all the phages, with various length and adjuvanticity display similar colloidal stability.

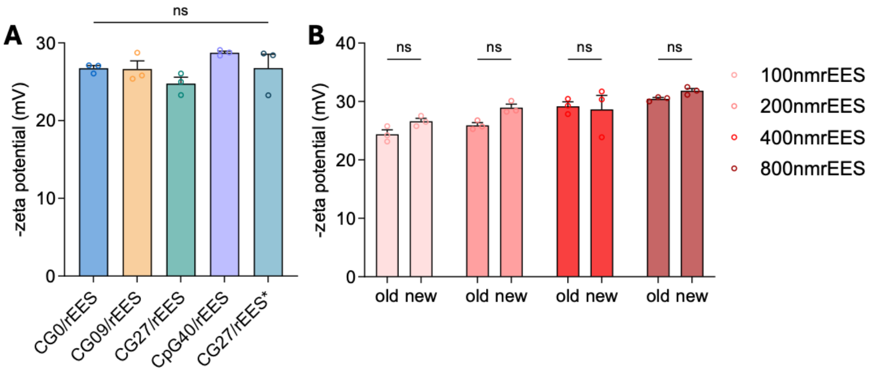

Fig. S6. RP phages of various length and adjuvanticity exhibit similar zeta potential and stability. (**A**) Phages of various adjuvanticity and (**B**) length demonstrate similar zeta potential and stability over time. The data show mean ± s.e.m.. ns, not significant, analyzed by one-way ANOVA (**A**) or two-way ANOVA (**B**) with Bonferroni post hoc test. The phages were dispersed in 1× PBS solution diluted by 20 times when performing the zeta potential measurement.

Fig. S7. Longer phages exhibited slower lymph node drainage and lower DC uptake

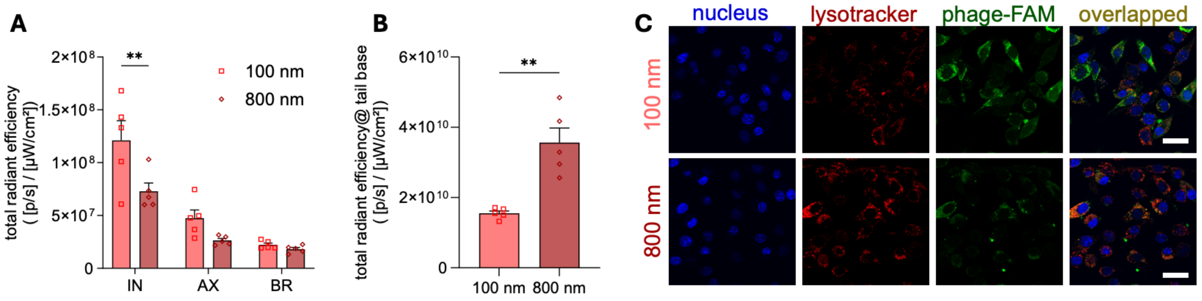

Figure S7. 800 nm phages demonstrated slower lymph node drainage and dendritic cell uptake than 100 nm phages. 100 nm phages demonstrated (**A**) higher lymph node drainage efficacy and (**B**) faster drainage kinetics and (**C**) higher dendritic cell uptake than 800 nm phages. Scale bar represents 10 µm in (**B**). **Fig. S7A** and **B** belonged to two different study cohorts. The data show mean ± s.e.m.. **p<0.01, analyzed by two-way ANOVA (**A**) with Bonferroni post hoc test or Student's t test (**B**).

100 nm and 800 nm phages with antigen pVIII (SIINFEKL) display ratio of 13.6% were administered via subcutaneous injection at the tail base with a dose of 5×10^{12} phage particles (calculated based on ssDNA base of 7234). 24 hours post injection, the inguinal, axially and brachial lymph nodes were excised to measure the fluorescence with IVIS and the results indicated that 100 nm phages demonstrated higher lymph node drainage efficacy than the 800 nm phages (Fig. S7A). As expected, the 800 nm phages demonstrated higher injection site retention that the 100 nm phages (Fig. S7B).

To compare the cellular uptake efficiency of the two phages, DC2.4 cells were seeded in a 96-well plate at a seeding density of 10000 cells/well overnight and then 10^{12} phage particles (calculated based on ssDNA base of 7234) were added. Lysotracker dye was used to assess the colocalization between the phages and the late endosome/lysosomes. After 48 hours of incubation with phages, the phage uptake study indicated that 800 nm phages accumulated less in DC2.4 cells compared to the 100 nm phages (Fig. S7C). In addition, both of the two phages were captured by the lysosome and displayed similar intracellular distribution.

Fig. S8. Shorter RP phages exhibited better immune activation at lower antigen density

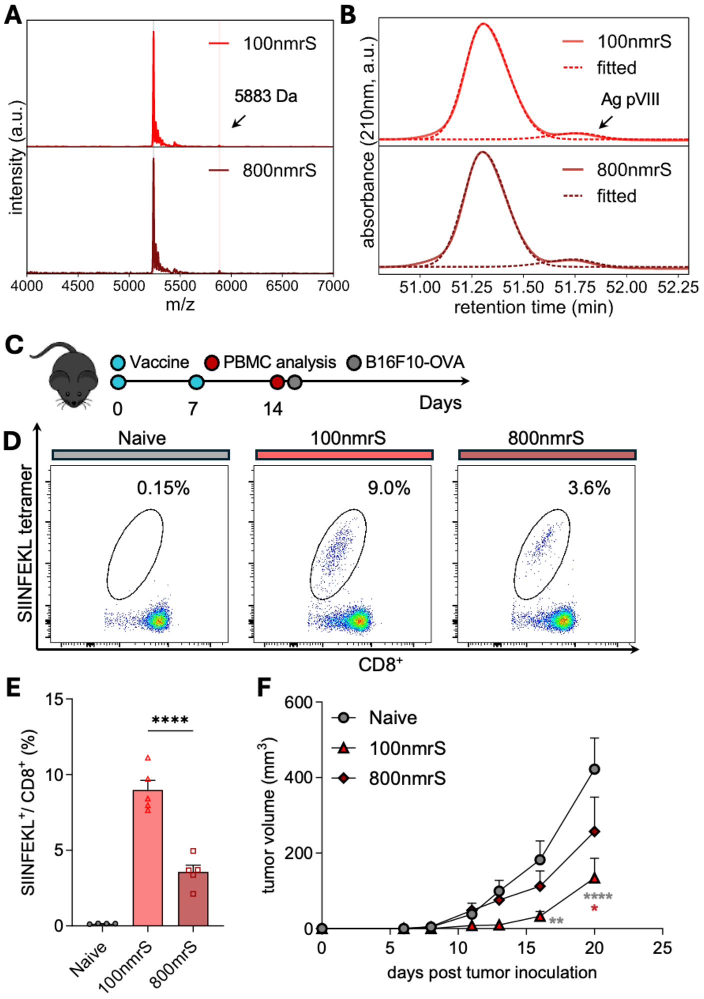

Fig. S8. 100 nm RP phages demonstrated higher antigen-specific CD8$^+$ T cell response at lower antigen density. (**A**) Confirmation of the antigen pVIII display at 5883Da with MALDI-TOF. (**B**) HPLC quantified the antigen pVIII display ratio to be ~ 6% for both the 100 nm and 800 nm phages. Following the in vivo vaccination schedule in (**C**) 5×10^{12} phage particles (calculated based on ssDNA base of 7234) were administered subcutaneously at tail base on day 0 and 7. The 100 nm phages elicited higher SIINFEKL-specific CD8$^+$ T cell frequency than the 800 nm phages, as indicated by the representative FACS plots in (**D**), and the summary in (**E**). (**F**) 100 nm phages demonstrated better tumor prevention efficacy compared to the 800 nm phages when challenged with 0.2M B16F10-OVA cells subcutaneously. The data show mean ± s.e.m.. *p<0.05, **p<0.01, ***p<0.001 and ****p<0.0001, analyzed by one-way ANOVA (**E**) or two-way ANOVA (**F**) with Bonferroni post hoc test.

In Fig. 5I and J of the main text, phages of different lengths elicited similar SIINFEKL-specific CD8$^+$ T cell response and similar tumor prevention efficacy. To further investigate the length effect, we amplified phages of 100 nm and 800 nm with lower antigen display in combination with the rS helper plasmid. As studied with MALDI-TOF (Fig. S8A) and HPLC (Fig. S8B), ~ 6% of the antigen pVIII at 5583Da was displayed on the phage capsid. Following the vaccination schedule in Fig. S8C, the in vivo vaccination study demonstrated that the 100 nm phages elicited higher SIINFEKL-specific CD8$^+$ T cell response compared to the 800 nm phages (9.0% vs 3.6%) (Fig. S8D and E), and better tumor prevention (Fig. S8F). In summary, shorter phages demonstrate better vaccine efficacy at lower antigen density.

Fig. S9. FACS gating of the antigen-specific CD8$^+$ memory T cells

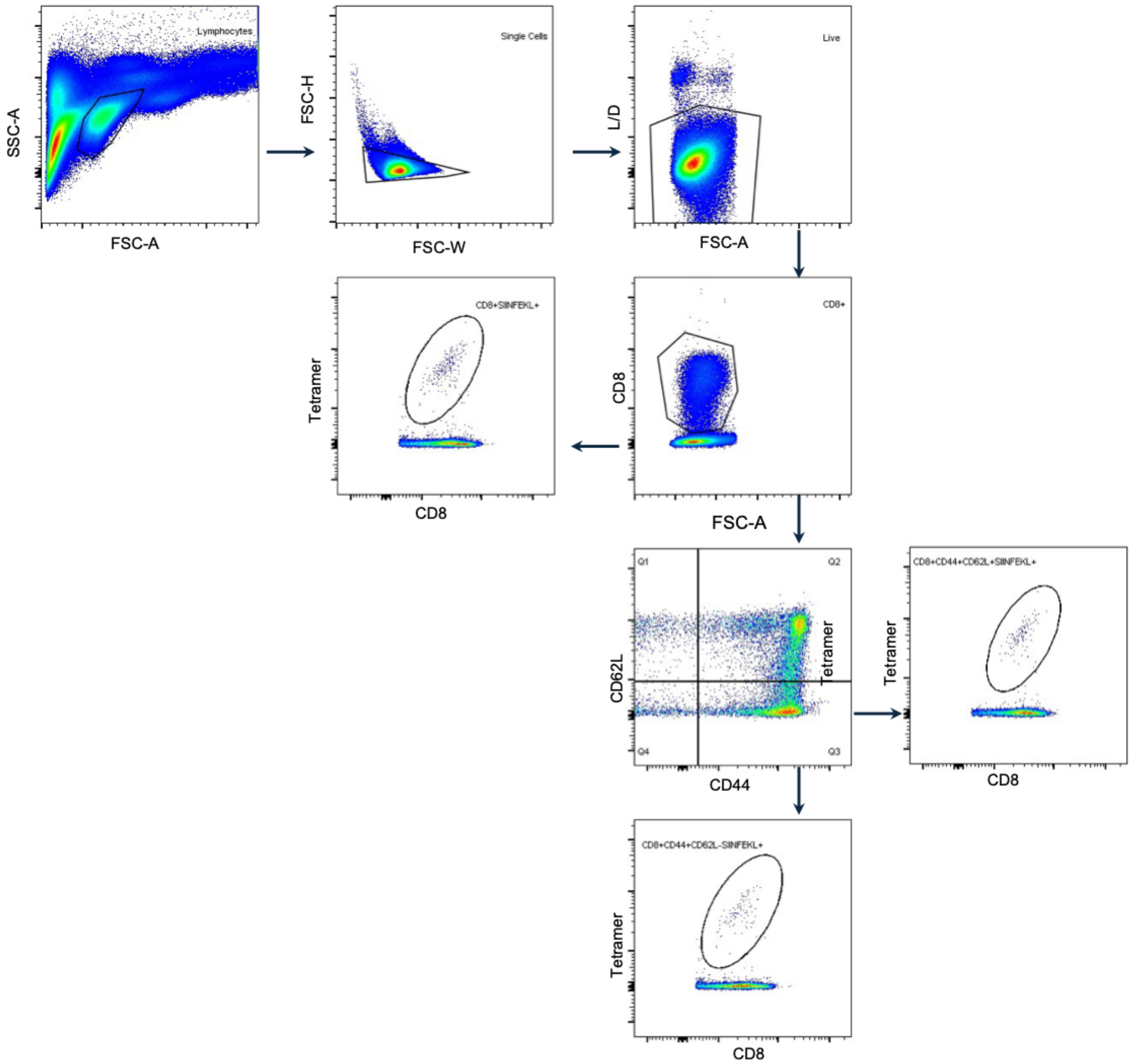

Fig. S10. M13 phages demonstrated superior biocompatibility towards mammalian cells

DC2.4 cells were seeded in a 96-well plate overnight and then incubated with up to 10^{12} RP phages/ well. The viability of the DC2.4 cells were evaluated with the viability staining (L3976, LIVE/DEAD Fixable Near-IR Dead Cell Stain Kit) using flow cytometry post 6 hours, 24 hours and 48 hours incubation. Specifically, the results demonstrated that the phages had little impact on the viability of the dendritic cells, indicating the high safety and superior biocompatibility of the phages toward mammalian cells.

In addition, 24 hours after immunization with RP phages, no signs of splenomegaly or systemic inflammation were observed relative to the naïve mice.

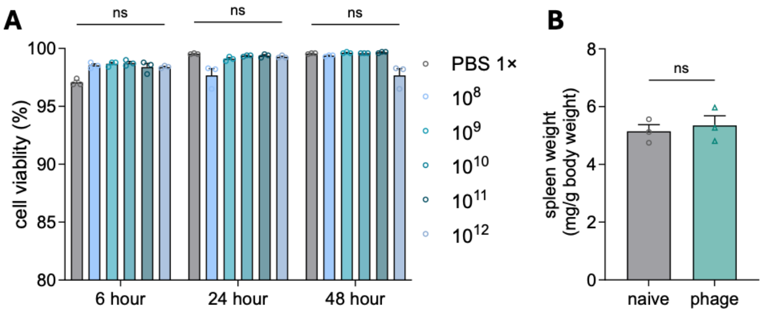

Fig. S10. RP phages demonstrated superior biocompatibility. (**A**) Viability of the DC2.4 cells at different RP phage (CG27/rEES) doses. (**B**) Quantification of spleen/body weight ratios of mice 24 hours after vaccination. The data show mean ± s.e.m.. ns, not significant, analyzed by two-way ANOVA (**A**) with Bonferroni post hoc test or Student's t test (**B**).

Fig. S11. cDNA sequencing of the mouse Adpgk gene

The total RNA of MC-38 cells was extracted using the RNeasy Mini Kit (74104, Qiagen). The first-strand cDNA was synthesized using 5μg of the total RNA with the SuperScript III First-Strand Synthesis SuperMix (18080400, Invitrogen). Adpgk cDNA with lengths of 555 bp were amplified using the forward primer:5'-CTGGAGGTGTTTGTGTCTAG-3' and reverse primer 5'-TCTTAGAGACACTCGGTTGG-3' with the Q5® High-Fidelity 2× Master Mix (New England Biolabs). The length of the PCR product was confirmed with 2% agarose gel and the sequence was confirmed with Sanger sequencing using the forward primer as the sequencing primer.

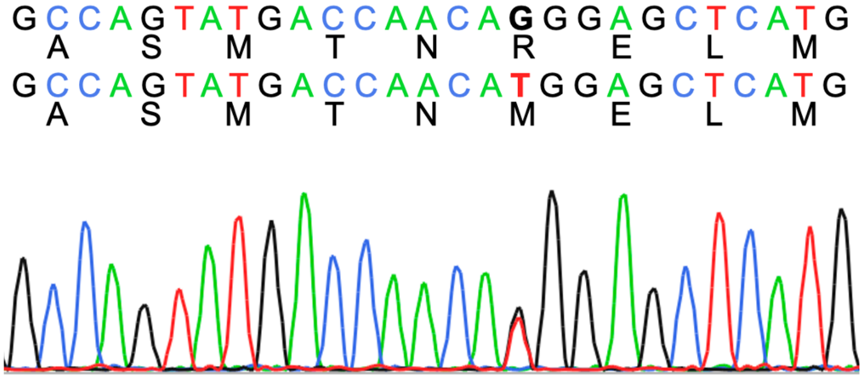

Fig. S11. cDNA sequencing of the mouse Adpgk gene shows the Adpgk mutation (ASMTNRELM → ASMTNMELM).

Fig. S12. HPLC collection and MALDI-TOF MS analysis of the Adpgk RP phage

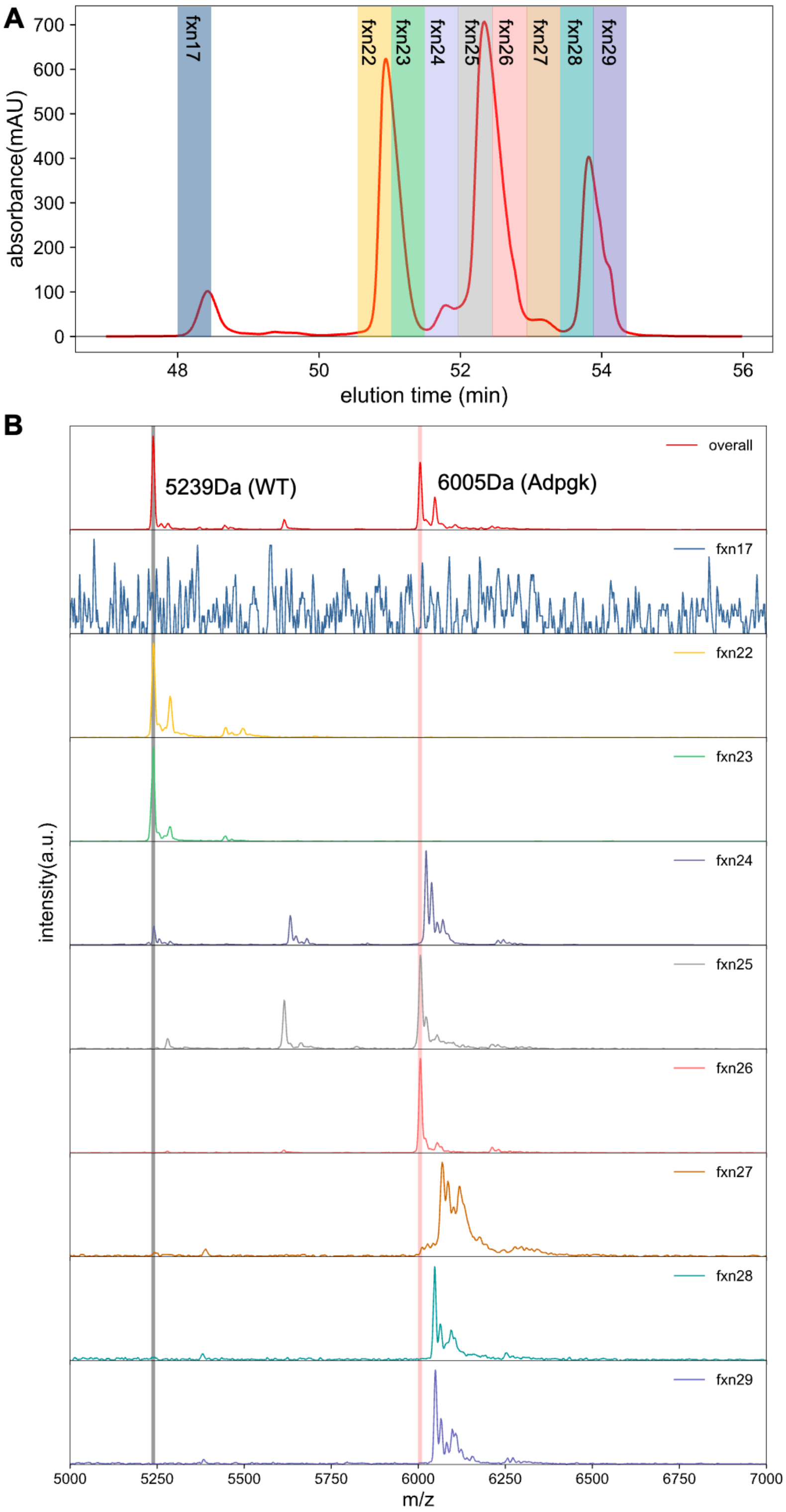

Fig. S12. Identifying the Adpgk pVIII peak with HPLC and MALDI-TOF MS. (**A**) HPLC collection plot of the Adpgk phage with the shading areas indicating the fractions (fxn) that were collected for MALDI-TOF analysis. (**B**) MALDI-TOF analysis of the collected fractions in (**A**). The results show that fractions 22,23 and 25,26 were wild-type pVIII (5239Da) and the Adpgk pVIII (6005Da), respectively.

Fig. S13. Low neoantigen-specific CD8$^+$ T cell response of the Adpgk RP phages due to low neoantigen display ratio

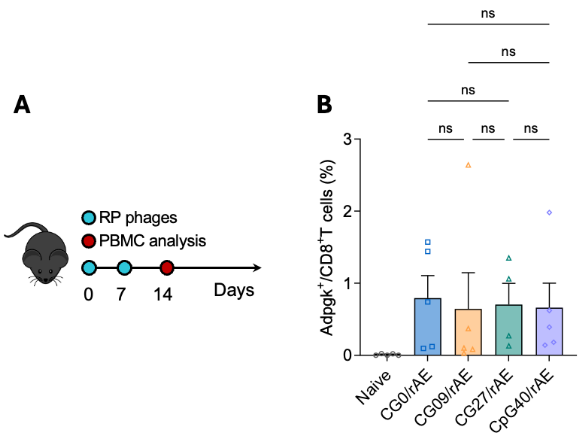

Fig. S13. Initial trial with Adpgk pVIII-displaying RP phages showed low immune response. (**A**) Dosing schedule of the initial vaccination study with the RP phages amplified by combining different RP phagemids and the rAE helper plasmid. (**B**) The resultant low neoantigen-specific CD8$^+$ T response as studied by the tetramer staining. ns: not significant, analyzed by one-way ANOVA (**B**) with Bonferroni post hoc test.

Fig. S14. Treatment timing is critical for combination therapy outcomes

In the course of evaluating combination therapy, we identified the complicated dependency of therapeutic efficacy on the treatment timing. Specifically, for the same tumor inoculation dose, early-stage treatment starting at day 8, when the average tumor size was ~ 45 mm^3, yielded a complete regression rate exceeding 80% (Fig. S14, A to C). In contrast, administration at a later stage of day 10, when the average tumor size was ~ 80 mm^3, substantially diminished therapeutic outcomes (Fig. S14, D to F). These findings emphasize the importance of early tumor diagnosis and highlight the challenges associated with managing advanced malignancies.

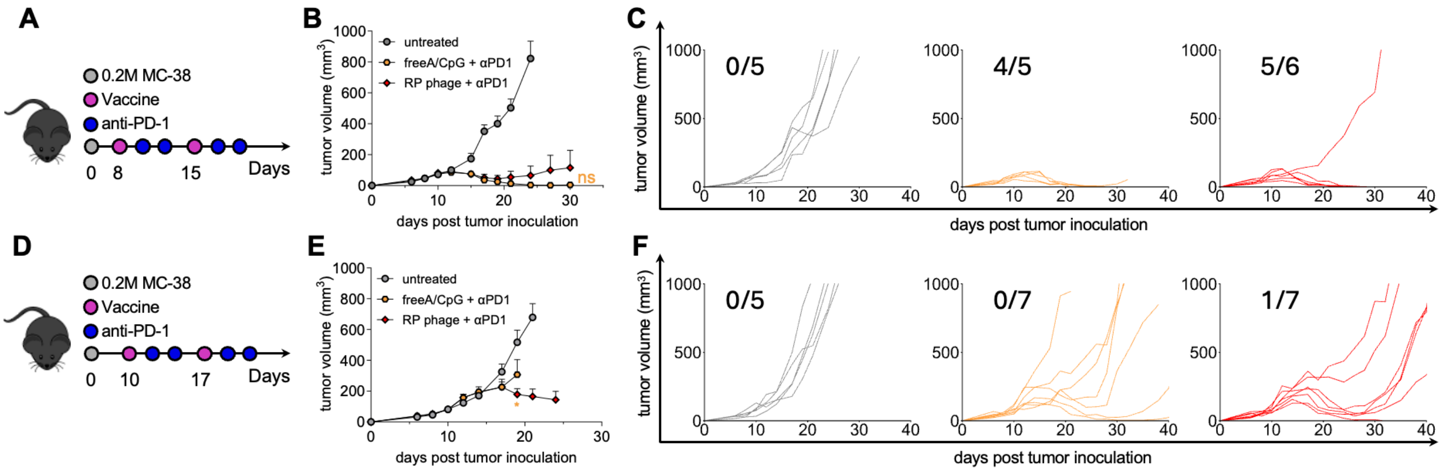

Fig. S14. Combination therapy efficacy depends strongly on the treatment timing. (**A** to **C**) Combination treatment started at day 8 yielded high complete regression rate, with 0.2 M MC-38 inoculation when the average tumor volume was ~ 45 mm^3. (**A**) RP phages (7.5×10^{12} phage particles calculated based on a ssDNA length of 7234 nucleotides, 100 μL 1× PBS) or equivalent combinations of free Adpgk peptide (~ 23.7 μg) and CpG (~ 5.4 nmol) were administered in two doses on day 8 and 15 post tumor inoculation. Anti-mouse PD-1 antibodies (100 μg) were administered on day 2 and 4 following each vaccination treatment. (**B**) Average tumor volume of the combined therapy study and (**C**) individual MC-38 tumor growth curves. (**D** to **F**) Combination treatment started at day 10 yielded low complete regression rate, with 0.2 M MC-38 inoculation when the average tumor volume was ~ 80 mm^3. (**D**) RP phages (7.5×10^{12} phage particles calculated based on a ssDNA length of 7234 nucleotides, 100 μL 1× PBS) or equivalent combinations of free Adpgk peptide (~ 23.7 μg) and CpG (~ 5.4 nmol) were administered in two doses on day 10 and 17 post tumor inoculation. Anti-mouse PD-1 antibodies (100 μg) were administered on day 2 and 4 following each vaccination treatment. (**E**) Average tumor volume of the combined therapy study and (**F**) individual MC-38 tumor growth curves. ns: not significant, $*p<0.05$, analyzed by two-way ANOVA (**B**,**E**) with Bonferroni post hoc test.

Fig. S15. Mouse body weight measurement during the treatment

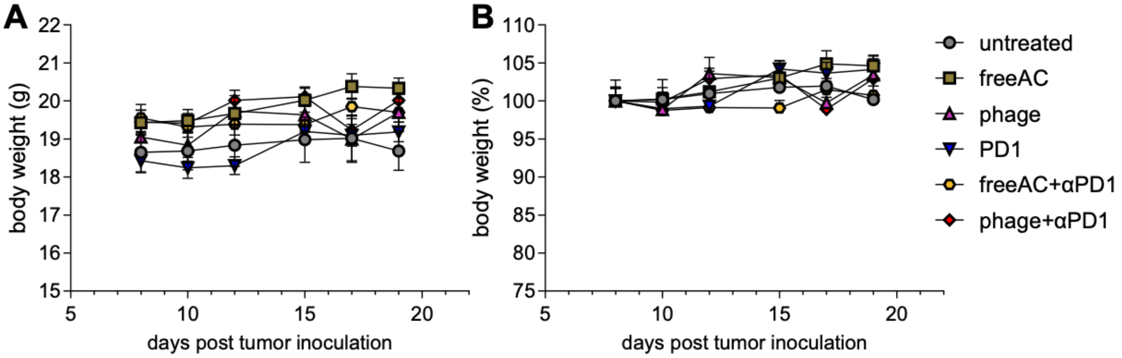

Fig. S15. Mouse body weight of different study groups changed little during the course of treatment. (**A**) Absolute body weight and (**B**) relative body weight normalized to the average body weight of each group at day 8 just before the treatment.

Fig. S16. Robust memory immunity induced by combination therapy

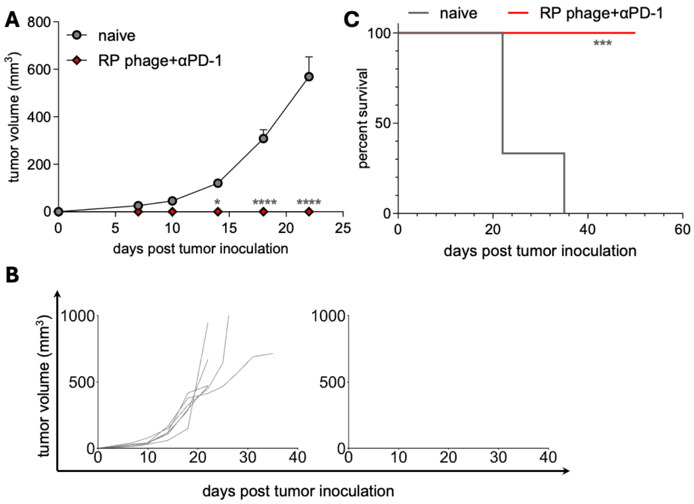

Fig. S16. Robust memory immunity induced by the combination therapy of the RP phage-based nanovaccine and αPD-1 antibody. (**A**) Average tumor volume, (**B**) individual tumor volume and (**C**) survival curve of the age-matched naïve mice and the mice survived the combination therapy. 0.1M MC-38 cells were inoculated subcutaneously around 5 months after the combination therapy. ns: not significant, $*p<0.05$, $***p<0.001$, $****p<0.0001$, analyzed by two-way ANOVA (**A**) with Bonferroni post hoc test, and log rank test (**C**).

To evaluate the memory immunity induced by the combination therapy of the RP phage-based nanovaccine and αPD-1 antibody, the 6 mice that survived the combination therapy (Fig. 8F-K) were rechallenged with 0.1M MC-38 tumor cells about 5 months after the treatment, together with another 6 age-matched naïve mice. As shown in Fig. S16, all the mice in the naïve groups developed tumors and succumbed to the tumor burden with a median survival time of 22 days, on the contrary, none of the mice that survived the combination therapy developed tumors 50 days post the tumor challenge, suggesting the robust memory immunity from the combination therapy.

Table S1. Reprogrammed ssDNA sequence of the RP phages

Name	Sequence (5'→3')
CG0	aatagtggactcttgttccaaactggaacaacactcaaccctatctcgggctattcttttgatttataagggattttgccgatttcggcctattggttaaa<br>aaatgagctgatttaacaaaaatttaacgcgacatgtTGATATGCCTAAGGCAAGTTGCCATGTGCTGTGGGCT<br>ATCAGGCAGATTAGCTCTGATGCTCTAGCAGAGGCCATAGCAATAGGCATTTGCTGGAGG<br>CATCAGCCAAGGCCAGGTGCCAAAGGCCCCTTGCCAGGAGCCAGTGGCCTTACAGCAAT<br>GAGCATCTTTGCTAGCTGCCTTTGTGCCATAGGCTAGGGCATGGAGCTACAGGCAAGAG<br>AGCTTTCTAGCTCCCAGCCAATTTGCTTCAAGCACCTGCCATCAAGCTATTGCAAACAGG<br>CCTTTGGGCTTTCCAGCACACTGCAGACTTGCCTTGAGCCTACATGCATGCTGCCCCAGC<br>ACTGTGCCAGAAGCAGGTTTGCATGTCAGCTTAGTGCATATCAGCTTTGGCCATGAGCAC<br>CTTGCTCTGTAGCAGAAGAGCATCTAGGCATTTGGCAATAGTGCTCTGAGCCAAATGCTG<br>CTATGCTGCACTGCCTGTGGCAACAGGCTTAAGAGCCCTTGAGCTTGGCAGCACCAGTG<br>CTTTTGGCAATTGCAACTAGGCCAAGAGGCAAGTCAGCCAGCCAGCAGATCAGCTAGCT<br>AGCAGTAGGCATGTGCTACTGTGCAAAATGGCTAAAAAGCCACTGGGCCAGGGGCTTAA<br>CTGCACAAGGGCTAAGGAGCTAAAAGCTCTATGCTCATGCAGAGAAGCTATGCTGCTCT<br>GGGCTTGTTGCCCTCTGCACCTGGGCTTGGGGCTTGATGCATTAGGCTCTAGGCTGAAGG<br>CTCAGAGCTGGACAGCCTCCTGGCATTAAGCTTGAGTGCATAGGGCACCTGGCTAACTA<br>GCATGGTGCAGGAGGCCTAGTAGCATTGGCTAATAGGCAGGATGCTTATGCATATAGCAT<br>GGTGGCTGTATGCATGTGCAGACAGCATTAGTGCTCAGGAGCAAAGGGCTACCTGCCCA<br>ATGGCCAACTGCTTGTAGCCACCAAGCATTCAGGCgcggccgcacgcgatacaatccgcgccctgtagcggc<br>gcattaagcgcggcgggtgtggtggttacgcgcagcgtgaccgctacacttgccagcgccctagcgcccgctcctttcgctttcttcccttcctttc<br>tcgccacgttcgccggctttccccgtcaagctctaaatcgggggctccctttagggttccgatttagtgctttacggcacctcgaccccaaaaaact<br>tgatttgggtgatggttcacgtagtgggccatcgccctgatagacggtttttcgccctttgacgttggagtccacgttcttt
CG09	aatagtggactcttgttccaaactggaacaacactcaaccctatctcgggctattcttttgatttataagggattttgccgatttcggcctattggttaaa<br>aaatgagctgatttaacaaaaatttaacgcgacatgtTGATATCGCTAAGCGAAGTTGCCATGTGCTGTGGGCT<br>ATCAGGCAGATTAGCTCTGATCGTCTAGCAGAGCGCATACGAATAGGCATTTGCTGGAGG<br>CATCAGCCAAGGCCAGGTGCCAAAGGCCCCTTGCCAGGAGCCAGTGCGCTTACACGAAT<br>GAGCATCTTTGCTAGCTGCCTTTGTGCCATAGCGTAGGCGATGGAGCTACAGGCAAGAG<br>ACGTTTCTAGCTCCCACGCAATTTCGTTCAAGCACCTCGCATCAACGTATTGCAAACAGC<br>GCTTTGGGCTTTCCACGACACTCGAGACTTGCCTTGACGCTACATCGATGCTGCCCCAGC<br>ACTGTCGCAGAACGAGGTTTGCATGTCAGCTTAGTCGATATCAGCTTTGGCCATGAGCAC<br>CTTCGTCTGTAGCAGAAGAGCATCTAGGCATTTGGCAATAGTGCTCTGAGCCAAATGCTG<br>CTATGCTGCACTGCCTGTGCGAACAGGCTTAAGAGCCCTTGAGCTTGGCACGACCAGTG<br>CTTTTGGCAATTGCAACTAGCGCAAGAGCGAAGTCAGCCAGCCACGAGATCACGTAGCT<br>AGCAGTAGGCATGTGCTACTGTGCAAAATGGCTAAAAAGCCACTGGGCCAGGGCGTTAA<br>CTCGACAAGGCGTAAGGACGTAAAAGCTCTATGCTCATGCAGAGAAGCTATGCTCGTCT<br>GGGCTTGTTGCCCTCTGCACCTGGCGTTGGGCGTTGATGCATTAGCGTCTAGCGTGAAGG<br>CTCAGAGCTGGACACGCTCCTGGCATTAAGCTTGAGTCGATAGGGCACCTGGCTAACTA<br>CGATGGTGCAGGAGGCCTAGTAGCATTGCGTAATAGGCAGGATGCTTATGCATATAGCAT<br>GGTGGCTGTATGCATGTGCAGACAGCATTAGTGCTCAGGACGAAAGGGCTACCTCGCCA<br>ATGGCCAACTCGTTGTAGCCACCAAGCATTCAGGCgcggccgcacgcgatacaatccgcgccctgtagcggc<br>gcattaagcgcggcgggtgtggtggttacgcgcagcgtgaccgctacacttgccagcgccctagcgcccgctcctttcgctttcttcccttcctttc<br>tcgccacgttcgccggctttccccgtcaagctctaaatcgggggctccctttagggttccgatttagtgctttacggcacctcgaccccaaaaaact<br>tgatttgggtgatggttcacgtagtgggccatcgccctgatagacggtttttcgccctttgacgttggagtccacgttcttt
CG27	aatagtggactcttgttccaaactggaacaacactcaaccctatctcgggctattcttttgatttataagggattttgccgatttcggcctattggttaaa<br>aaatgagctgatttaacaaaaatttaacgcgacatgtTGATATCGCTAAGCGAAGTTCGCATGTCGTGTGGCGT<br>ATCAGCGAGATTACGTCTGATCGTCTACGAGAGCGCATACGAATAGCGATTTCGTGGAGC<br>GATCACGCAAGCGCAGGTCGCAAAGCGCCCTTCGCAGGACGCAGTGCGCTTACACGAA<br>TGACGATCTTTCGTAGCTCGCTTTGTCGCATAGCGTAGGCGATGGACGTACAGCGAAGAG<br>ACGTTTCTACGTCCCACGCAATTTCGTTCAACGACCTCGCATCAACGTATTCGAAACAGC<br>GCTTTGGCGTTTCCACGACACTCGAGACTTCGCTTGACGCTACATCGATGCTCGCCCACG<br>ACTGTCGCAGAACGAGGTTTCGATGTCACGTTAGTCGATATCACGTTTGCGCATGACGAC<br>CTTCGTCTGTACGAGAAGACGATCTAGCGATTTGCGAATAGTCGTCTGACGCAAATCGTG<br>CTATCGTGCACTCGCTGTGCGAACAGCGTTAAGACGCCTTGACGTTGGCACGACCAGTC

	GTTTTGCGAATTCGAACTAGCGCAAGAGCGAAGTCACGCAGCCACGAGATCACGTAGCT<br>ACGAGTAGCGATGTCGTACTGTCGAAAATGCGTAAAAACGCACTGGCGCAGGGCGTTAA<br>CTCGACAAGGCGTAAGGACGTAAAACGTCTATCGTCATCGAGAGAACGTATGCTCGTCT<br>GGCGTTGTTCGCCTCTCGACCTGGCGTTGGGCGTTGATCGATTAGCGTCTAGCGTGAAGC<br>GTCAGACGTGGACACGCTCCTGCGATTAACGTTGAGTCGATAGGCGACCTGCGTAACTA<br>CGATGGTCGAGGAGCGCTAGTACGATTGCGTAATAGCGAGGATCGTTATCGATATACGAT<br>GGTGCGTGTATCGATGTCGAGACACGATTAGTCGTCAGGACGAAAGGCGTACCTCGCCA<br>ATGCGCAACTCGTTGTACGCACCAACGATTCAGCGgcggccgcacgcgatacaatccgcgccctgtagcggc<br>gcattaagcgcggcgggtgtggtggttacgcgcagcgtgaccgctacacttgccagcgccctagcgcccgctcctttcgctttcttcccttcctttc<br>tcgccacgttcgccggctttccccgtcaagctctaaatcgggggctccctttagggttccgatttagtgctttacggcacctcgaccccaaaaaact<br>tgatttgggtgatggttcacgtagtgggccatcgccctgatagacggtttttcgccctttgacgttggagtccacgttcttt
CpG40	aatagtggactcttgttccaaactggaacaacactcaaccctatctcgggctattcttttgatttataagggattttgccgatttcggcctattggttaaa<br>aaatgagctgatttaacaaaaatttaacgcgacatgtTGATATCGCTAAGGACGTTAAGTTCGCATGTGACGTTT<br>GTGGCGTATCAGCGAGATTAGACGTTTCTGATAACGTTTCTACGAGAGGACGTTCATACG<br>AATAGGACGTTATTTCGTGGAGCGATCAGACGTTCAAGAACGTTCAGGTCGCAAAGCGC<br>CCTTGACGTTCAGGAAACGTTCAGTGCGCTTACACGAATGACGATCTTTCGTAGCTCGCT<br>TTGTAACGTTCATAGCGTAGGGACGTTATGGACGTACAGAACGTTAAGAGACGTTTCTAG<br>ACGTTTCCCAGACGTTCAATTTCGTTCAACGACCTCGCATCAAAACGTTTATTCGAAACA<br>GCGCTTTGGCGTTTCCACGACACTCGAGACTTCGCTTGAGACGTTCTACATCGATGCTAA<br>CGTTCCCAGACGTTACTGTGACGTTCAGAACGAGGTTTCGATGTCACGTTAGTCGATATC<br>AGACGTTTTTGGACGTTCATGACGACCTTAACGTTTCTGTACGAGAAGACGATCTAGCGA<br>TTTGGACGTTAATAGTCGTCTGAGACGTTCAAATCGTGCTATCGTGCACTGACGTTCTGT<br>GGACGTTAACAGCGTTAAGACGCCTTGACGTTGGCACGACCAGTCGTTTTGGACGTTAA<br>TTCGAACTAGCGCAAGAGCGAAGTCACGCAGCCAAACGTTAGATCACGTAGCTAGACGT<br>TAGTAGGACGTTATGTCGTACTGTCGAAAATGCGTAAAAACGCACTGGCGCAGGGCGTT<br>AACTGACGTTACAAGGCGTAAGGACGTAAAACGTCTATCGTCATCGAGAGAACGTATGC<br>TGACGTTTCTGGCGTTGTTAACGTTCCTCTCGACCTGGCGTTGGGGACGTTTTGATCGAT<br>TAGCGTCTAGGACGTTTGAAGCGTCAGAGACGTTTGGACACGCTCCTGCGATTAACGTT<br>GAGTAACGTTATAGGGACGTTACCTGCGTAACTAGACGTTATGGTCGAGGAGCGCTAGTA<br>GACGTTATTGCGTAATAGCGAGGATAACGTTTTATGACGTTATATAAACGTTATGGTGCGT<br>GTATCGATGTCGAGACAAACGTTATTAGTCGTCAGGAGACGTTAAAGGGACGTTTACCTG<br>ACGTTCCAATGAACGTTCAACTCGTTGTAGACGTTCACCAACGATTCAGCGgcggccgcacgc<br>gatacaatccgcgccctgtagcggcgcattaagcgcggcgggtgtggtggttacgcgcagcgtgaccgctacacttgccagcgccctagcgc<br>ccgctcctttcgctttcttcccttcctttctcgccacgttcgccggctttccccgtcaagctctaaatcgggggctccctttagggttccgatttagtgct<br>ttacggcacctcgaccccaaaaaacttgatttgggtgatggttcacgtagtgggccatcgccctgatagacggtttttcgccctttgacgttggagtc<br>cacgttcttt

*gBlock is highlighted in capital letters.

Table S2. Controlling the CG fraction in the RP phages of varying length

Table S2.1. ssDNA sequence of the RP phages of varying length

ssDNA base number/phage length	Sequence (5’→3’)
721/100 nm	aatagtggactcttgttccaaactggaacaacactcaaccctatctcgggctattcttttgatttataagggattttgccgatttcggcctattggttaaaaaatgagctgatttaacaaaaatttaacgcgacatgtATTACGTTAACGACGTTTCGAATCGAGAACGTCATCGATTCGCTGCGCTACGTCTCGACTGCGCTAGCGTATCGTGCGTAGCGATACGATCACGAAGCGTTAGCGTGTACGAACTCGCTCGTACGATTTCGTCACGTTCGCACGACCTCGTCGTGAGCGACACGAACGCAACGATCGAGCGCCTACGTTACGTGCTCGCTTGCGTGACGATCTCGCCTCGTGAACGTGGCGAGTTCGCTTCGCAGACGACAACGAGGCGCAGCGTTGGCGgcggccgcacgcgatacaatccgcgccctgtagcggcgcattaagcgcggcgggtgtggtggttacgcgcagcgtgaccgctacacttgccagcgccctagcgcccgctcctttcgctttcttcccttcctttctcgccacgttcgccggctttccccgtcaagctctaaatcgggggctccctttagggttccgatttagtgctttacggcacctcgaccccaaaaaacttgatttgggtgatggttcacgtagtgggccatcgccctgatagacggtttttcgccctttgacgttggagtccacgttcttt
1447/200 nm	aatagtggactcttgttccaaactggaacaacactcaaccctatctcgggctattcttttgatttataagggattttgccgatttcggcctattggttaaaaaatgagctgatttaacaaaaatttaacgcgacatgtATTACGTTAACGACGTTTCGTTCGTGAGCGATAGGCGACCCACGATGGGCGAAAGGGCGAATCACGTGGGCTCGATATACGCAGAGGCGTGTACGAACTACGAGAACGTAACGCCCTTCGTTCTACGTATCCACGACAAAGCGTTGGTCGTAGCTCGAACACTCGTTAAGGCGCAGATCGTCCTTCGAGCCTCGTAGGTGCGAGATCGTTAGCGCTGGGACGATATTCGTAACTCGAGGTGCGTTCCTCGTGGTTCGAGGCTGCGAGTCTTCGTACCACGATATCGCCTTGCGCATTTCGTGTCGAATAACGTACTGACGATTCTCGAGCTAACGTCATCGTCCAGACGTTGCAGCGTACCTCGCATAGCGCAAAACGCAAAAGCGAGACACGATCTGACGATGCACGAGCTCGATTGGCGCCCTCGTTCAAGCGCAAGTCGCAAAGCGAGTGTGCGTCACACGTATGCGAGTATCGCCCAAGCGTCATTACGATGACACGCAACTGCGCTCCACGTCCAACGTAAATTCGCACATCGTGCAGCGATAGGGCGAGATGCGCTGTGCGAATAGCGTCACGAACATCGCTAGTCGTCTTTCGTGCAATCGAGGAGCGAGCACGCCTATCGCATTTGCGTTGGCGTAACACGTTACTCGCTTGCGTTCATCGCTGGGCGCTTTTCGTGCTGCGTTTGTCGCTGTCGCCTGTCGAGTTACGTGTTCACGTCAAGCGCCTTCGCAGTAACGAAATCTCGTAATCGCAGGGGCGTTCAGCGAGGTCACGCTTCACGTTTAACGAGAAGCGTACAATCGTACTCTCGTCAGCGACCTTACGCAACTCGCAATAACGTTCTCGCCAGTGCGCACTTGCGTCCTCGTTGTTACGCTCACTCGAGGCACGTACATTCGAGAGACGCTCTTCGTCTAGCGCATAGTCGACTGAGCGTAATTCGCAAACTCGAACAACGTCCAATCGCAACGAGGCGCAGCGTTGGCGGCggccgcacgcgatacaatccgcgccctgtagcggcgcattaagcgcggcgggtgtggtggttacgcgcagcgtgaccgctacacttgccagcgccctagcgcccgctcctttcgctttcttcccttcctttctcgccacgttcgccggctttccccgtcaagctctaaatcgggggctccctttagggttccgatttagtgctttacggcacctcgaccccaaaaaacttgatttgggtgatggttcacgtagtgggccatcgccctgatagacggtttttcgccctttgacgttggagtccacgttcttt
3241/400 nm	aatagtggactcttgttccaaactggaacaacactcaaccctatctcgggctattcttttgatttataagggattttgccgatttcggcctattggttaaaaaatgagctgatttaacaaaaatttaacgcgacatgtATTACGTTAACGACGTTTCGAACTGTCGTGATTACGTCCTTGCGTATCACGTAGTGTCGTTTGTCGTGCTGCGTAGTAACGAAGGAACGTAGGGGCGATTCAACGATTTTCGTCACACGATTTGCGCAAAAGCGTAACTACGCCATCGTCCAAACGTCTCTCGTGGCACGTAGCAGCGCATTCTCGAACTATCGTCCTGCGTAATGACGTGAAAGCGAGCAACGTATATACGTAGGGCGACCCTGCGCTAGTCGCTGGTTCGTGCTGTCGTACTGCGTTTCGAATGCACGTACCACGTCCACACGTTAACTCGCTTATCGAGCATCGAGAGGGCGAGACCACGTAGACTCGAATCTTCGTTCCCTCGTGATTTCGCAAAGGCGTAGGGACGCTATGCGCTTAATCGTGAGGCGTACAGCGAGCATGCGACCCTACGAGCTCGACTCTCGTTTTGCGACTGGCGAGGTACGAGCCAACGCTGTTCGATTCACGAATGATCGCCTATGCGCTAGTACGCACTTTCGTTGGACGCAGGACGTCAAGCGCATGATCGTTTGTGCGAGACTCGCATGGCGAGCTGGCGTCTGACGCCCAACGTCTCTACGTTAGATCGATGCCACGCAAATACGCCTGGACGATAATCGTCTTATCGTGAAGCGATGCCTCGCTACCTCGAGTGTCGTATGTCGAATCACGAAGAAACGCCCCAGCGAGTGACGTATCCTCGCTATTGCGCATTCGATAGATCGCTGAGCGTCCTATCGACATATCGCCCAAACGTAGCACGTCCTACGTCCCTCGCTCTTCGCCAAGTCGCTGGAGCGCTGTGTCGCCTGTTCGTAGTGCGCTTTGACGCTTTACGATTAGCGACTAAACGTTTTCACGCACTCTCGCAGCACGAGTTGACGCAAGGCGTGGTTACGAAGGTCGAAAATGCGAGGAGTCGTGATGGCGCCTAGGCGCTCCATCGCACTGTCGACTCTGCGTTTCCTCGCAGACACGAATTAACGCCTATGCGTTGAACGTACAGCGTGGATCGAAGGGCGATGTACGACTCTCGAGACTACGCCATACGCTGTGTCGCTCTAT

	CGACTGATCGAGGAAGCGCTTAGCGTAATTCGTGATTACGACCTCGTGTCCTCGATGCCTCG<br>ATAATTCGTATATACGCAATGCGAACTGACGCATTTGCGTCTTCGCTACGAACATCGAAAAGT<br>CGTAAATACGTTCCCACGATTATCGCTACATCGATTTTCGATTTAGCGTGTCAACGATCCAGC<br>GATAGGCGCCAGGCGTTAGAGCGTACAAACGTGTTCACGCATAACGCCTTACGATATTACGA<br>GTGAACGAGAAAGCGTGAAACGAGGCTTCGCACTACGAGCCACGTCCTTTCGCTAGACGA<br>TTACTCGTTTACTCGCTGGACGTAGATCGAATAAGCGTTTAGACGAATTGCGTATTGGCGATC<br>CACGAACTTACGCCTAACGCTGTTGCGTATAGCGCTTTGCGTTCAGCGTGTAGCGCCTTTCG<br>AGGATCGCATGCGAATCTACGAAAGCACGTGCTTGCGTTGGGCGTGTAGTCGTGACACGAC<br>TAACGTAGGTCGAGCATCGAAGTACGCATATGCGTCCTGCGTAAAGCGCTTCTACGAGATAG<br>CGAGCCAACGCTTCACGCCATTACGCAAGTACGTGCCTACGAGCTACGATGATGCGATGAAT<br>CGAGTATGCGTTAAGCGTCCCAACGTCAGTCGAGAAACGTGGGATCGAACTCACGTATTGA<br>CGACTTTCGCTGGTCGCCACAGCGATAAACGCATAGTCGAAGTTACGACAACACGCCCTGC<br>GCTAACGTCCTTCGCCACAACGAAAATTCGCTGTTGCGCCTAGGCGCTTGTCGTTTACGCC<br>ATGCGTCAAAACGTCTGTCGCCTATTCGTGTGGACGCTGTGCGACTAACGTCACAGCGAGT<br>TTCGCCCCATCGACTACACGTACCTCGCAGGGGCGTCATTCGAGCAACGATGTGCGTTCTGG<br>CGAAAGGCGTCAATCGCCATAGCGACTATACGTCCACGTCAACTCGCTGTCACGCAGATCG<br>CCACTTCGATAAACGATTTACGTGGGACGTGAAGCGATTTAGCGTCCTGACGCCAAGCGAA<br>TTTGCGCAAGGGCGTCATAACGAAATCGAAGCAGCGTCCTCTCGCTTCATCGCAGAACGTG<br>TTCTCGATGTTTCGTTAGGCGCTGGACGTTACTCGCAGCGATGAGCGCCCTGCGAGCATCGT<br>TATACGCAGGTACGAATTGCGAAGCCACGCTGCCTCGTGTACTCGCTGCACGTTCAGCGTGT<br>ATTCGCACATACGCAATAGCGTGCTATCGCAATTCGCTTCTCGCTCTTCGTCAACGCATTGCG<br>CTTCACGCCTTCACGACATATCGCTAAAGCGAAGACGTCAAGCGTTGCCACGAAGCTCGAA<br>GAATCGCCCAGACGTCCTTTCGAGACACGAGATTCGTAAACACGACATCACGTCTGATCGC<br>AGAGGCGTGGCTACGAAAAGCGAATAGCGCTAGACGTCCATCGAGGGACGCAGTAACGAA<br>CTTCGCTGCTCGTTGTTCGTAGGACGCTGATACGAGGGGCGTGCATGCGTTTGCGAAACAG<br>CGTAGTTTCGAATGGCGTTTGTCGCCCCACGAGTGTCGACCTGCGTAAATACGAAAAGACG<br>AGTAGCGAAAGTACGCTCATTCGAGGAAGCGATGTGTCGAATATCGAACAAACGAAATTGC<br>GCTTAACGCCTGCTCGTGTCCCGAGGCGCAGCGTTGGCGGCggccgcacgcgatacaatccgcgccctgtag<br>cggcgcattaagcgcggcgggtgtggtggttacgcgcagcgtgaccgctacacttgccagcgccctagcgcccgctcctttcgctttcttcccttccttt<br>ctcgccacgttcgccggctttccccgtcaagctctaaatcgggggctccctttagggttccgatttagtgctttacggcacctcgaccccaaaaaacttg<br>atttgggtgatggttcacgtagtgggccatcgccctgatagacggtttttcgccctttgacgttggagtccacgttcttt
6261/800 nm	aatagtggactcttgttccaaactggaacaacactcaaccctatctcgggctattcttttgatttataagggattttgccgatttcggcctattggttaaaaaa<br>tgagctgatttaacaaaaatttaacgcgacatgtATTACGTTAACGACGTTTCGAATCGAGAACGTCATCGATTCG<br>CTGCGCTACGTCTCGACTGCGCTAGCGTATCGTGCGTAGCGATACGATCACGAAGCGTTAGC<br>GTGTACGAACTCGCTCGTACGATTTCGTCACGTTCGCACGACCTCGTCGTGAGCGACACGA<br>ACGCAACGATCGAGCGCCTACGTTACGTGCTCGCTTGCGTGACGATCTCGCCTCGTGAACG<br>TGGCGAGTTCGCTTCGCAGACGACAATTCGTGAGCGATAGGCGACCCACGATGGGCGAAA<br>GGGCGAATCACGTGGGCTCGATATACGCAGAGGCGTGTACGAACTACGAGAACGTAACGCC<br>CTTCGTTCTACGTATCCACGACAAAGCGTTGGTCGTAGCTCGAACACTCGTTAAGGCGCAG<br>ATCGTCCTTCGAGCCTCGTAGGTGCGAGATCGTTAGCGCTGGGACGATATTCGTAACTCGAG<br>GTGCGTTCCTCGTGGTTCGAGGCTGCGAGTCTTCGTACCACGATATCGCCTTGCGCATTTCG<br>TGTCGAATAACGTACTGACGATTCTCGAGCTAACGTCATCGTCCAGACGTTGCAGCGTACCT<br>CGCATAGCGCAAAACGCAAAAGCGAGACACGATCTGACGATGCACGAGCTCGATTGGCGC<br>CCTCGTTCAAGCGCAAGTCGCAAAGCGAGTGTGCGTCACACGTATGCGAGTATCGCCCAAG<br>CGTCATTACGATGACACGCAACTGCGCTCCACGTCCAACGTAAATTCGCACATCGTGCAGC<br>GATAGGGCGAGATGCGCTGTGCGAATAGCGTCACGAACATCGCTAGTCGTCTTTCGTGCAAT<br>CGAGGAGCGAGCACGCCTATCGCATTTGCGTTGGCGTAACACGTTACTCGCTTGCGTTCATC<br>GCTGGGCGCTTTTCGTGCTGCGTTTGTCGCTGTCGCCTGTCGAGTTACGTGTTCACGTCAAG<br>CGCCTTCGCAGTAACGAAATCTCGTAATCGCAGGGGCGTTCAGCGAGGTCACGCTTCACGT<br>TTAACGAGAAGCGTACAATCGTACTCTCGTCAGCGACCTTACGCAACTCGCAATAACGTTCT<br>CGCCAGTGCGCACTTGCGTCCTCGTTGTTACGCTCACTCGAGGCACGTACATTCGAGAGAC<br>GCTCTTCGTCTAGCGCATAGTCGACTGAGCGTAATTCGCAAACTCGAACAACGTCCAATCGC

	AATCGACACAGCGACCTACGTCCCAACGTCCTCACGCAGACACGCTTGAGCGATGCACGAT<br>GAGTCGTTCTTCGACCAACGATTAACGCTTATCGTCTTTCGCTTAGGCGTTCTACGTGTTGCG<br>CAACTGCGAGGGGCGTGCTGCGACAGCACGTGATGACGTTGCCTCGTATTACGAAGTAACG<br>TAAGTTCGAGAAGTCGTGATTTCGTTAAGGCGCACTACGATGGGGCGATAGATCGAAGATAC<br>GTGTACTCGCAGTTCGAGAGCGACAATTCGTCTAGCGCTCCATCGAGGTGCGACTTAACGAT<br>ACACGTAGGGCGCTTTGCGTTATGCGCTGGACGCAACCACGTAATTCGCTATACGAGGTCGT<br>TGAAACGACAGATCGACTTAGCGCCAGACGTAAGAGCGAAAGGCGCAGCGAGTCTCGCTA<br>ACGATGGACGAACAGCGATTACTCGCCACAACGCTAGGGCGATTAGCGCATGTCGTTCTGT<br>CGTAGAGGCGATTATCGATCCTGCGCTAAACGTTGTGCGAGACACGTAAAACGCTAGATCGT<br>GATTGCGAGCTTTCGCCAGATCGAAGGACGTGATGGCGAAGTACGAGCTCTCGCTTGGCGA<br>AACAACGAGGAGCGTGTTATCGATGGTCGCAGATCGCCAACTCGTGTGAACGCTAATCGCT<br>TCTGCGCACCTGCGTGTAGTCGTCCCACGAAGGTTCGTGCTCGAATGCGTACACGCTGAGT<br>CGCTGGTCGAGGCTTCGCAGTCTCGTTGGGCGAAATTCGCAAAGACGAGTTTCGACTCTAC<br>GTAAGTCGTAACAACGTGTCTCGACTCTCGCCATTTCGAAGGCACGTGAACTCGATGTGCG<br>TGGTGCGTGTGGCGTCAAAACGAATCACGATTTCGTACTGCGTTTCACGAATTAGCGACCAT<br>CGTTGAACGTCGAATATCGTGAATCGTTTACACGCAAAGCGTACCTTCGCTCATTCGAGTGT<br>CGACAGACGAATCACGCCTATCGCAAAGCGATATACGTAAGCGATAGTTCGTTGAACGAAAT<br>GCGAGTCCTCGTAAGCTCGAGATGTCGCTCACACGAGGTAACGTTGAGGCGTAGATGCGTG<br>TGAACGCATAGCGCTCAAACGAACTTCGTTGAAGCGTGGATGCGCTCCCACGTTCCAACGA<br>AGCTGCGTGGGACGATGATGCGAGCAGTCGTTTTAACGTCAGGTCGATTCTTCGTGGAACG<br>AGGTACGATGGGCGTCCAGCGTTCCATCGAACTACGAATGCTCGTCACTTCGACTGGCGAC<br>CTCACGCATGAACGCTAGACGCTGGCACGCTCAGCGTATAAACGTTAGGTCGAAAATGCGA<br>TCCACGCCATGCGACCCACGAATCATCGATTGTACGATGGGGCGTAGGACGACTCTTCGAAT<br>CAACGATCTTCGTGATACGATTAACGAGAAGTCGATATGACGACTTGTCGCAACCTCGCTTG<br>GACGACCTAGCGATAGACGTTTTCGAGTTCGTCCTGCGTTTCTCGAGGATCGTTGGGCGAG<br>CATTCGCACAACGATGAGCGCCAGACGCAGATCGAAGCAACGCTATACGACACTGCGACTC<br>ACGCCCTGCGTATTGTCGCAGTATCGAAGATCGACCCTCGTGCCATCGTCTCACGAGTCATC<br>GCATAGCGTGGATCGAAGCCACGTTAAGCGTCCCTGCGCCCTGCGCCAAACGCTAAACGCT<br>GGTTCGTAGATCGTTGCTACGAGTTCTCGCTATTACGATGTACGATCAACGAGAGCGTTAAA<br>CGCCTTACGAAAATACGAAGACACGAATAGCGTCACCTCGCAACTGCGTTCAACTGTCGTG<br>ATTACGTCCTTGCGTATCACGTAGTGTCGTTTGTCGTGCTGCGTAGTAACGAAGGAACGTAG<br>GGGCGATTCAACGATTTTCGTCACACGATTTGCGCAAAAGCGTAACTACGCCATCGTCCAA<br>ACGTCTCTCGTGGCACGTAGCAGCGCATTCTCGAACTATCGTCCTGCGTAATGACGTGAAAG<br>CGAGCAACGTATATACGTAGGGCGACCCTGCGCTAGTCGCTGGTTCGTGCTGTCGTACTGCG<br>TTTCGAATGCACGTACCACGTCCACACGTTAACTCGCTTATCGAGCATCGAGAGGGCGAGA<br>CCACGTAGACTCGAATCTTCGTTCCCTCGTGATTTCGCAAAGGCGTAGGGACGCTATGCGCT<br>TAATCGTGAGGCGTACAGCGAGCATGCGACCCTACGAGCTCGACTCTCGTTTTGCGACTGG<br>CGAGGTACGAGCCAACGCTGTTCGATTCACGAATGATCGCCTATGCGCTAGTACGCACTTTC<br>GTTGGACGCAGGACGTCAAGCGCATGATCGTTTGTGCGAGACTCGCATGGCGAGCTGGCGT<br>CTGACGCCCAACGTCTCTACGTTAGATCGATGCCACGCAAATACGCCTGGACGATAATCGTC<br>TTATCGTGAAGCGATGCCTCGCTACCTCGAGTGTCGTATGTCGAATCACGAAGAAACGCCCC<br>AGCGAGTGACGTATCCTCGCTATTGCGCATTCGATAGATCGCTGAGCGTCCTATCGACATATC<br>GCCCAAACGTAGCACGTCCTACGTCCCTCGCTCTTCGCCAAGTCGCTGGAGCGCTGTGTCG<br>CCTGTTCGTAGTGCGCTTTGACGCTTTACGATTAGCGACTAAACGTTTTCACGCACTCTCGC<br>AGCACGAGTTGACGCAAGGCGTGGTTACGAAGGTCGAAAATGCGAGGAGTCGTGATGGCG<br>CCTAGGCGCTCCATCGCACTGTCGACTCTGCGTTTCCTCGCAGACACGAATTAACGCCTATG<br>CGTTGAACGTACAGCGTGGATCGAAGGGCGATGTACGACTCTCGAGACTACGCCATACGCT<br>GTGTCGCTCTATCGACTGATCGAGGAAGCGCTTAGCGTAATTCGTGATTACGACCTCGTGTC

	CTCGATGCCTCGATAATTCGTATATACGCAATGCGAACTGACGCATTTGCGTCTTCGCTACGA<br>ACATCGAAAAGTCGTAAATACGTTCCCACGATTATCGCTACATCGATTTTCGATTTAGCGTGT<br>CAACGATCCAGCGATAGGCGCCAGGCGTTAGAGCGTACAAACGTGTTCACGCATAACGCCT<br>TACGATATTACGAGTGAACGAGAAAGCGTGAAACGAGGCTTCGCACTACGAGCCACGTCCT<br>TTCGCTAGACGATTACTCGTTTACTCGCTGGACGTAGATCGAATAAGCGTTTAGACGAATTG<br>CGTATTGGCGATCCACGAACTTACGCCTAACGCTGTTGCGTATAGCGCTTTGCGTTCAGCGT<br>GTAGCGCCTTTCGAGGATCGCATGCGAATCTACGAAAGCACGTGCTTGCGTTGGGCGTGTA<br>GTCGTGACACGACTAACGTAGGTCGAGCATCGAAGTACGCATATGCGTCCTGCGTAAAGCG<br>CTTCTACGAGATAGCGAGCCAACGCTTCACGCCATTACGCAAGTACGTGCCTACGAGCTACG<br>ATGATGCGATGAATCGAGTATGCGTTAAGCGTCCCAACGTCAGTCGAGAAACGTGGGATCG<br>AACTCACGTATTGACGACTTTCGCTGGTCGCCACAGCGATAAACGCATAGTCGAAGTTACG<br>ACAACACGCCCTGCGCTAACGTCCTTCGCCACAACGAAAATTCGCTGTTGCGCCTAGGCGC<br>TTGTCGTTTTACGCCATGCGTCAAAACGTCTGTCGCCTATTCGTGTGGACGCTGTGCGACTA<br>ACGTCACAGCGAGTTTCGCCCCATCGACTACACGTACCTCGCAGGGGCGTCATTCGAGCAA<br>CGATGTGCGTTCTGGCGAAAGGCGTCAATCGCCATAGCGACTATACGTCCACGTCAACTCGC<br>TGTCACGCAGATCGCCACTTCGATAAACGATTTACGTGGGACGTGAAGCGATTTAGCGTCCT<br>GACGCCAAGCGAATTTGCGCAAGGGCGTCATAACGAAATCGAAGCAGCGTCCTCTCGCTTC<br>ATCGCAGAACGTGTTCTCGATGTTTCGTTAGGCGCTGGACGTTACTCGCAGCGATGAGCGCC<br>CTGCGAGCATCGTTATACGCAGGTACGAATTGCGAAGCCACGCTGCCTCGTGTACTCGCTGC<br>ACGTTCAGCGTGTATTCGCACATACGCAATAGCGTGCTATCGCAATTCGCTTCTCGCTCTTCG<br>TCAACGCATTGCGCTTCACGCCTTCACGACATATCGCTAAAGCGAAGACGTCAAGCGTTGC<br>CACGAAGCTCGAAGAATCGCCCAGACGTCCTTTCGAGACACGAGATTCGTAAACACGACAT<br>CACGTCTGATCGCAGAGGCGTGGCTACGAAAAGCGAATAGCGCTAGACGTCCATCGAGGGA<br>CGCAGTAACGAACTTCGCTGCTCGTTGTTCGTAGGACGCTGATACGAGGGGCGTGCATGCG<br>TTTGCGAAACAGCGTAGTTTCGAATGGCGTTTGTCGCCCCACGAGTGTCGACCTGCGTAAA<br>TACGAAAAGACGAGTAGCGAAAGTACGCTCATTCGAGGAAGCGATGTGTCGAATATCGAAC<br>AAACGAAATTGCGCTTAACGCCTGCTCGTGTCCCGAGGCGCAGCGTTGGCGgcggccgcacgcgat<br>acaatccgcgccctgtagcggcgcattaagcgcggcgggtgtggtggttacgcgcagcgtgaccgctacacttgccagcgccctagcgcccgctc<br>ctttcgctttcttcccttcctttctcgccacgttcgccggctttccccgtcaagctctaaatcgggggctccctttagggttccgatttagtgctttacggcac<br>ctcgaccccaaaaaacttgatttgggtgatggttcacgtagtgggccatcgccctgatagacggtttttcgccctttgacgttggagtccacgttcttt

Table S2.2. CG fractions of the RP phages of varying length

ssDNA base number	phage length	CG dimer number	*CG fraction (%)
721	100 nm	91	25.2
1447	200 nm	181	25.0
3241	400 nm	414	25.5
6261	800 nm	839	26.8

CG fraction is calculated as: 2×CG dimer number/ssDNA base number

Table S3. Summary of the helper plasmids developed in this work

Table S3.1. Helper plasmid naming and antigen sequence

Helper plasmid name	Helper plasmid construct	Antigen sequence	Short name for antigen sequence	Regulatory mutation for antigen pVIII expression	Expression of the antigen peptide at native or recombinant pVIII
rEES	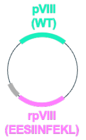	**EES**IINFEKL	EES	No	recombinant
EES	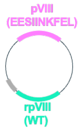	**EES**IINFEKL	EES	No	native
EESmut	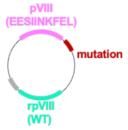	**EES**IINFEKL	EES	Yes	native
rAE	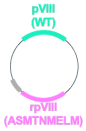	**ASMTNMELME**	AE	No	recombinant
AE	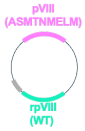	**ASMTNMELME**	AE	No	native

Table S3.2. Full amino acid sequence of the pVIII

helper plasmid	location	pVIII type	mature pVIII sequence
rEES	recombinant pVIII	antigen pVIII	AEESIINFEKL[#]DPAKAAFDSLQASATEYIGYAW AMVVVIVGATIGIKLFKKFTSKAS
	native pVIII	wild-type pVIII	AEGDDPAKAAFDSLQASATEYIGYAWAMVVVIV GATIGIKLFKKFTSKAS
EES	native pVIII	antigen pVIII	AEESIINFEKLDPAKAAFDSLQASATEYIGYAWAM VVVIVGATIGIKLFKKFTSKAS
	recombinant pVIII	wild-type pVIII	AEGDDPAKAAFDSLQASATEYIGYAWAMVVVIV GATIGIKLFKKFTSKAS
EESmut	native pVIII	antigen pVIII	AEESIINFEKLDPAKAAFDSLQASATEYIGYAWA MVVVIVGATIGIKLFKKFTSKAS
	recombinant pVIII	wild-type pVIII	AEGDDPAKAAFDSLQASATEYIGYAWAMVVVIV GATIGIKLFKKFTSKAS
rAE	recombinant pVIII	antigen pVIII	ASMTNMELMEDPAKAAFDSLQASATEYIGYAW AMVVVIVGATIGIKLFKKFTSKAS
	native pVIII	wild-type pVIII	AEGDDPAKAAFDSLQASATEYIGYAWAMVVVIV GATIGIKLFKKFTSKAS

AE	native pVIII	antigen pVIII	ASMTNMELMEDPAKAAFDSLQASATEYIGYAW<br>AMVVVIVGATIGIKLFKKFTSKAS
	recombinant pVIII	wild-type pVIII	AEGDDPAKAAFDSLQASATEYIGYAWAMVVVIV<br>GATIGIKLFKKFTSKAS

#: antigen sequences underlined and highlighted in purple.

Table S3.3. Nucleotide sequence the selected pVIII (including the 20 nucleotides proceeding the start codon of the pVIII)

helper plasmids	location	pVIII type	Nucleotide sequence of the pVIII (5'⟶3', including 20 bases proceeding the start codon)
EES	native pVIII	antigen pVIII	CGTTTAATGGAAGCTTCCTCATG^AAAAAGTCTTTAGT<br>CCTCAAAGCCTCTGTAGCCGTTGCTACCCTCGTTCCG<br>ATGCTGTCTTTCGCTGCTGAGGAAAGTATAATCAACT<br>TTGAAAAACTGGATCCCGCAAAAGCGGCCTTTGACT<br>CCCTGCAAGCCTCAGCGACCGAATATATCGGTTATGC<br>GTGGGCGATGGTTGTTGTCATTGTCGGCGCAACTATC<br>GGTATCAAGCTGTTTAAGAAATTCACCTCGAAAGCAA<br>GCTGA
EESmut	native pVIII	antigen pVIII	CGTTTAATGGT*AGCTTCCTCATG^AAAAAGTCTTTAG<br>TCCTCAAAGCCTCTGTAGCCGTTGCTACCCTCGTTCC<br>GATGCTGTCTTTCGCTGCTGAGGAAAGTATAATCAAC<br>TTTGAAAAACTGGATCCCGCAAAAGCGGCCTTTGAC<br>TCCCTGCAAGCCTCAGCGACCGAATATATCGGTTATG<br>CGTGGGCGATGGTTGTTGTCATTGTCGGCGCAACTAT<br>CGGTATCAAGCTGTTTAAGAAATTCACCTCGAAAGCA<br>AGCTGA

*mutation underlined and highlighted in grey. ^start codon of pVIII underlined
